# The fatal trajectory of pulmonary COVID-19 is driven by lobular ischemia and fibrotic remodelling

**DOI:** 10.1016/j.ebiom.2022.104296

**Published:** 2022-10-04

**Authors:** Maximilian Ackermann, Jan C. Kamp, Christopher Werlein, Claire L. Walsh, Helge Stark, Verena Prade, Rambabu Surabattula, Willi L. Wagner, Catherine Disney, Andrew J. Bodey, Thomas Illig, Diana J. Leeming, Morten A. Karsdal, Alexandar Tzankov, Peter Boor, Mark P. Kühnel, Florian P. Länger, Stijn E. Verleden, Hans M. Kvasnicka, Hans H. Kreipe, Axel Haverich, Stephen M. Black, Axel Walch, Paul Tafforeau, Peter D. Lee, Marius M. Hoeper, Tobias Welte, Benjamin Seeliger, Sascha David, Detlef Schuppan, Steven J. Mentzer, Danny D. Jonigk

**Affiliations:** a Institute of Pathology and Molecular Pathology, Helios University Clinic Wuppertal, University of Witten/Herdecke, Germany; b Institute of Functional and Clinical Anatomy, University Medical Center of the Johannes Gutenberg-University Mainz, Germany; c Department of Respiratory Medicine, Hannover Medical School, Hannover, Germany; d Member of the German Center for Lung Research (DZL), Biomedical Research in Endstage and Obstructive Lung Disease Hannover (BREATH), Hannover, Germany; e Institute of Pathology, Hannover Medical School, Hannover, Germany; f Centre for Advanced Biomedical Imaging, University College London, UK; g Research Unit Analytical Pathology, Helmholtz Zentrum München - German Research Center for Environmental Health, Neuherberg, Germany; h Institute of Translational Immunology and Research Center for Immune Therapy, University Medical Center, Johannes Gutenberg University, Mainz, Germany; i Department of Diagnostic and Interventional Radiology, University Hospital Heidelberg, Heidelberg, Germany; j Member of the German Center for Lung Research (DZL), Translational Lung Research Center Heidelberg (TLRC), Heidelberg, Germany; k Department of Mechanical Engineering, University College London, UK; l Diamond Light Source, Oxfordshire, Oxford, UK; m Hannover Unified Biobank, Hannover Medical School, Hannover Medical School, Germany; n Nordic Bioscience Biomarkers and Research, Herlev, Denmark; o Institute of Medical Genetics and Pathology, University Hospital Basel, Basel, Switzerland; p Institute of Pathology, RWTH Aachen University Hospital, Aachen, Germany.; q Department of Thoracic Surgery, University Hospital Antwerp Edegem, Belgium; r Department of Cardiothoracic, Transplantation, and Vascular Surgery, Hannover Medical School, Germany; s Department of Cellular Biology and Pharmacology, Center for Translational Research, Florida International University, USA; t European Synchrotron Radiation Facility, Grenoble, France; u Institute of Intensive Care Medicine, University Hospital Zurich, Zurich, Switzerland; v Division of Gastroenterology, Beth Israel Deaconess Medical Center, Harvard Medical School, Boston, MA, United States; w Laboratory of Adaptive and Regenerative Biology, Harvard Medical School, Brigham & Women's Hospital, Boston, United States

**Keywords:** COVID-19, Intussusceptive angiogenesis, Fibrogenesis, Biomarkers

## Abstract

**Background:**

COVID-19 is characterized by a heterogeneous clinical presentation, ranging from mild symptoms to severe courses of disease. 9–20% of hospitalized patients with severe lung disease die from COVID-19 and a substantial number of survivors develop long-COVID. Our objective was to provide comprehensive insights into the pathophysiology of severe COVID-19 and to identify liquid biomarkers for disease severity and therapy response.

**Methods:**

We studied a total of 85 lungs (n = 31 COVID autopsy samples; n = 7 influenza A autopsy samples; n = 18 interstitial lung disease explants; n = 24 healthy controls) using the highest resolution Synchrotron radiation-based hierarchical phase-contrast tomography, scanning electron microscopy of microvascular corrosion casts, immunohistochemistry, matrix-assisted laser desorption ionization mass spectrometry imaging, and analysis of mRNA expression and biological pathways. Plasma samples from all disease groups were used for liquid biomarker determination using ELISA. The anatomic/molecular data were analyzed as a function of patients’ hospitalization time.

**Findings:**

The observed patchy/mosaic appearance of COVID-19 in conventional lung imaging resulted from microvascular occlusion and secondary lobular ischemia. The length of hospitalization was associated with increased intussusceptive angiogenesis. This was associated with enhanced angiogenic, and fibrotic gene expression demonstrated by molecular profiling and metabolomic analysis. Increased plasma fibrosis markers correlated with their pulmonary tissue transcript levels and predicted disease severity. Plasma analysis confirmed distinct fibrosis biomarkers (TSP2, GDF15, IGFBP7, Pro-C3) that predicted the fatal trajectory in COVID-19.

**Interpretation:**

Pulmonary severe COVID-19 is a consequence of secondary lobular microischemia and fibrotic remodelling, resulting in a distinctive form of fibrotic interstitial lung disease that contributes to long-COVID.

**Funding:**

This project was made possible by a number of funders. The full list can be found within the Declaration of interests / Acknowledgements section at the end of the manuscript.

**Research in context**

**Evidence before this study**

The pathomechanism by which SARS-CoV2 causes the fatal trajectory of pulmonary pathology COVID-19 still remains vague. Since the beginning of the COVID-19 pandemic the role of the pulmonary vasculature has emerged in SARS-CoV2-induced ARDS.

**Added value of this study**

This study contributes substantially to the current knowledge on the pathophysiology of severe COVID-19 throughout the course of the disease. We have identified the distinct development of a fibrotic morpho-molecular pattern of remodelling. In comparison to other fibrotic lung diseases and to influenza A, changes in COVID-19 are driven by secondary lobular microischemia and the prolonged blood vessel neo-formation by intussusceptive angiogenesis, suggesting that treatment strategies aiming at preventing microvascular thrombosis and tissue ischemia (such as early oxygen supplementation and judicious anti-coagulation) may have a beneficial effect in patients with severe COVID-19 and avoid subsequent fibrotic remodelling. Moreover, our identification of complementary plasma markers of early fibrotic remodelling could serve as predictors of disease severity and therapeutic response in COVID-19.

**Implication of all the available evidence**

Our findings suggest a compelling explanation for mosaic-like microischemia in the severe pathology, which reflects the vasculopathy affecting the secondary lobule and the interlobular septae. We hypothesize that plasma markers (e.g. (TSP2, GDF15, IGFBP7, Pro-C3) could detect the of early fibrotic changes in COVID-19.

Alt-text: Unlabelled box

## Introduction

Coronavirus disease 2019 (COVID-19) is caused by the severe acute respiratory distress coronavirus 2 (SARS-CoV-2) in humans.^1^ Even with infection due to the same virus strain, COVID-19 manifests with a wide range of disease severity, varying from very mild to life-threatening. In a series of 44,000 subjects with COVID-19 in China,^2^ the illness demonstrated three distinct clinical presentations: 81% had mild symptoms, 14% had severe symptoms including dyspnea, hypoxia, and lung parenchymal involvement on computed tomography scan, and 5% had respiratory failure or multi-organ dysfunction. A meta-analysis published in January 2021 described a case fatality rate of 45% in 57,420 adult patients with severe COVID-19 who received invasive mechanical ventilation.^3^ Comorbidities leading to severe COVID-19 disease include the expected risk factors of respiratory viruses such as pre-existent pulmonary disease and immune dysfunction, and additional conventional cardiovascular risk factors such as hypertension, diabetes, ischemic heart and peripheral vascular disease, renal failure, and obesity.^4^ Although there is considerable speculation about how these comorbidities contribute to a grave course of the infection,^5^ the underlying pathogenesis of severe COVID-19 disease remains unclear.

To clarify this, we studied autopsy specimens of patients who succumbed to severe COVID-19 and compared them to autopsy lungs from patients who succumbed to severe influenza A (A[H1N1]) — a strain similar to the 1918/1919 and 2009 influenza pandemics — and to lung explants from patients with end-stage interstitial lung diseases (ILD) as well as to uninfected healthy lung specimens, obtained from surgical size adjustment specimen during lung transplantation and from archived non-infected, non-tumor formalin-fixed paraffin-embedded (FFPE) tissue.

The data were structured as a natural time-series experiment: the anatomic and molecular data were analyzed as a function of the patients’ hospitalization time to reflect the progressive pathophysiology of severe COVID-19 unfolding over time. Since each patient represents only one time-point in disease evolution, we did our best to reduce the impact of confounding individual variables such as unique comorbidities and sequelae of idiosyncratic intensive care management, by as narrowly defining the inclusion criteria as was practical in an autopsy cohort.

## Methods

**Figure 1:**
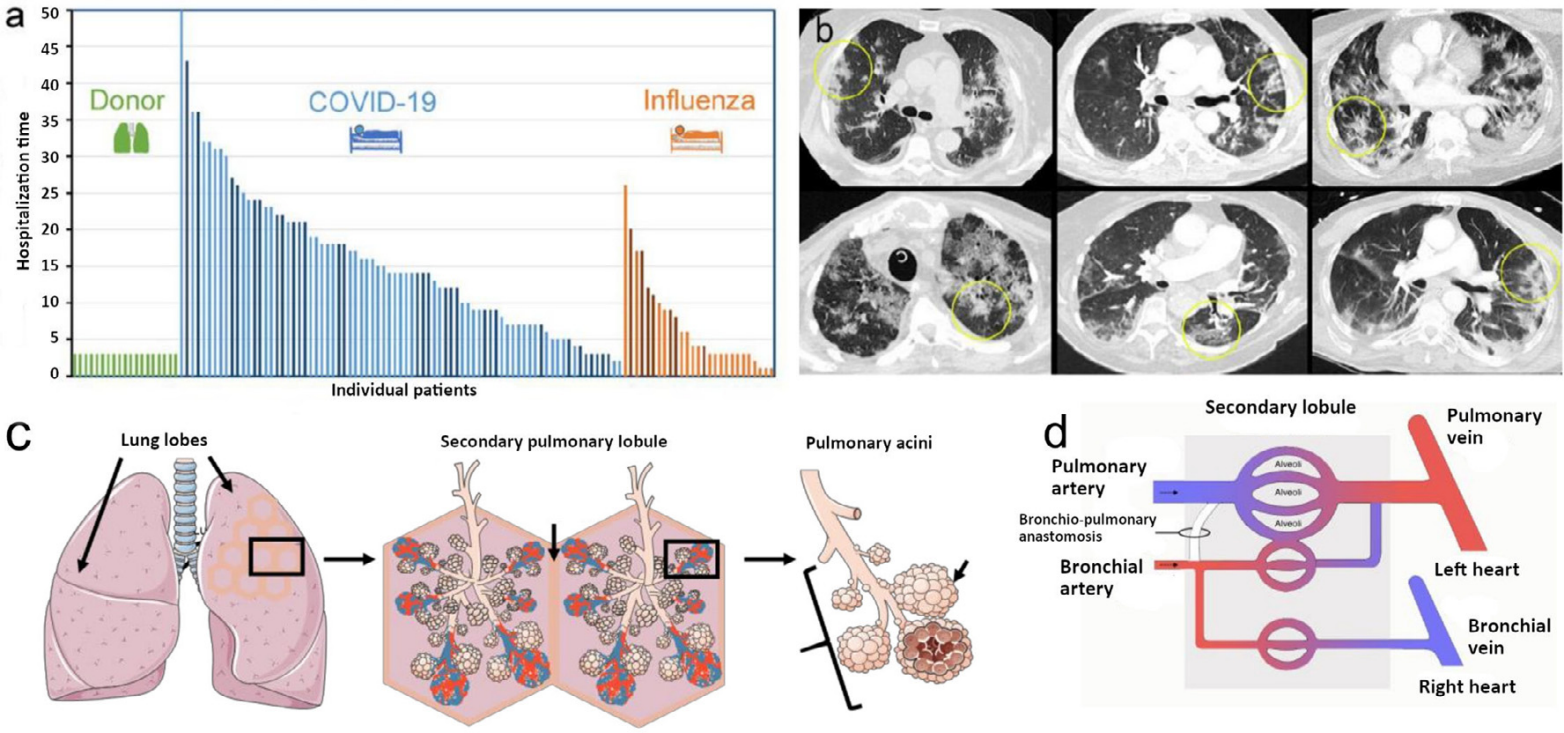
(a) Patient population with fatal COVID-19. Lung tissue and/or plasma samples were collected from 75 hospitalized COVID-19 patients, 17 hospitalized influenza A patients, and 19 healthy lung transplant donors. In most patients, serum and imaging studies were performed; a subset of patients was also analyzed via histopathology and gene expression analyses (dark blue and dark orange columns). (b) Dual energy computed tomography (CT) showed the characteristic features of severe COVID-19 pneumonia as bilateral ground-glass opacities, peribronchial consolidations, and a diffuse crazy-paving pattern characteristic of COVID-19 (yellow ellipses). (c) Illustration of anatomy and morphology of secondary lobules as an anatomical and functional subunit. (d) Schematic of pulmonary vascular supply consisting of pulmonary and bronchial circulation. The bronchial circulation primarily arises directly from the thoracic aorta and the intercostal arteries and constitutes less than 2% of the left cardiac output volume.

We analyzed pulmonary autopsy specimens from n = 31 patients who died from respiratory failure caused by severe COVID-19 and compared them with pulmonary autopsy specimens from n = 7 patients who died from pneumonia caused by influenza A virus subtype H1N1, n = 18 human lung explants from lung transplant recipients with different types of ILD (n = 6 usual interstitial pneumonia, UIP, n = 6 non-specific interstitial pneumonia, NSIP, n = 6 alveolar fibroelastosis, AFE) as well as with healthy lung tissue from n = 24 different donors (Figure 1a). In addition, we analyzed n = 81 plasma samples from hospitalized COVID-19 patients, clinically classified as mild, moderate, and severe, with follow-ups after convalescence, if applicable, and compared them to n = 20 plasma samples from hospitalized patients with influenza-related ARDS as well as to n = 17 plasma samples from ILD patients (IPF, n = 6; NSIP, n = 5; ILD with acute exacerbation, n = 6). All plasma samples were obtained from the Hannover Unified Biobank (HUB) and the ARDS registry of Hannover Medical School. Details on all included tissue and plasma samples are presented in the supplementary Tables E1-E2.

SARS-CoV-2 infections were confirmed by both ante-mortem nasal swabs and post-mortem lung PCR testing of FFPE lung tissue. Parenchymal infection was confirmed by mRNA expression analysis, demonstrating significant levels of viral RNAs encoding for 9 SARS-CoV-2 capsid proteins. All of the studied COVID-19 patients died of progressive respiratory failure secondary to the infection, sometimes in combination with bacterial superinfection and/or multi organ failure; COVID-19 patients with extracorporeal membrane oxygenation support or fatal comorbidities were excluded from the analysis. Patients who deceased within the first 7 days of hospitalization and patients who deceased later, were designated as early and late COVID-19 group, respectively.

The lungs from patients with influenza A were archived FFPE tissues from autopsies performed during the 2009 swine flu pandemic at Hannover Medical School. All samples were chosen for the best possible match with respect to age, sex, and disease severity. All ILD samples were obtained from fresh human lung explants at Hannover Medical School and the University of Leuven. Healthy lung tissues from surgically downsized or denied lung transplants as well as archived non-infected, non-tumor FFPE tissue were used as uninfected control specimens from Hannover Medical School and the University of Leuven. All diagnoses were made by experienced pulmonary pathologists according to the European Respiratory Society and American Thoracic Society guidelines.

### Ethics

The study was approved by and conducted according to the requirements of the ethics committees at the Hannover Medical School (no. 6921_BO_K_2021 and no. 2702–2015) and the University of Leuven (no. S52174). The study was performed in accordance with the declaration of Helsinki and regarding the rules and regulations of Lower Saxony. Written informed consent was obtained from all participants. The tissue samples from Leuven and influenza cases used in this study were the same as in a previous study.^6^ There was no commercial support for this study.

Lung tissues were multifariously analyzed using conventional histopathological assessment, immunohistochemical staining, Nanostring-based mRNA expression analysis, matrix-assisted laser desorption ionization (MALDI) mass spectrometry imaging, biological pathway analysis via the Qiagen Ingenuity pathway analysis tool, hierarchical phase-contrast tomography, micro-computed tomography, scanning electron microscopy of vascular corrosion casts, and Synchrotron radiation-based tomographic microscopy. Plasma samples were analyzed using validated enzyme linked immunosorbent assays (ELISA).

After fixation with 4% buffered formalin under a controlled inflation pressure of 55–65 cm H20 via the trachea, representative samples/sections of the lung were submitted for histological analysis. As remodelled lungs can contain a wide variety of injury patterns, patient, as well as control lungs, were systematically sampled and morphologically comparable areas systematically assessed by experienced pulmonary pathologists. According to established guidelines, the central and peripheral compartments of the upper, middle, and lower lungs were analyzed, examining the airways and vasculature, including capillaries as well as pre-and post-capillary vessels by Hematoxylin & Eosin, Elastica van Giesson, and Periodic Acid Schiff staining, while excluding any areas of necrosis. Histopathological features of COVID-19, influenza A, and ILD lungs were scored semi-quantitatively with regard to their site of manifestation. For qualitative and quantitative assessment of vascular alterations by conventional histology, stains were scanned at high magnification.

For immunohistochemistry, 2 µm thick slices of formalin-fixed, paraffin-embedded (FFPE) tissue were de-paraffinized with xylene for 10 min twice and rehydrated using decreasing ethanol concentrations. After heat-induced epitope retrieval in antibody buffer, sections were stained with primary antibodies according to the respective datasheets supplied by the manufacturer. Antibodies were visualized using the ZytoChem Plus HRP Polymer Kit (Zytomed systems) with DAB (3,3’-Diaminobinzidine) solutions. The detailed antibody list can be found in Table 1. Finally, slides were mounted and sealed using the Eukitt® mounting media. Images were recorded with the APERIO CS2 scanner (Leica Biosystems, Wetzlar, Germany) and processed using ImageScope software Version 12·3·3·5048 (Leica Biosystems, Wetzlar, Germany). All utilized antibodies have been validated as described in Figure E1 prior to their application in study samples.

**Table 1:** Antibodies used for immunostaining.

Target	Antibody	Pretreatment	Dilution
TGF-β1	Anti-TGF beta 1 antibody [TB21] (ab190503)	Citrate buffer (pH 6)	1:5000
HIF-1α	Anti-HIF-1 alpha antibody [ESEE122] (ab8366)	Tris-EDTA buffer (pH 9)	1:200
ANGPT-2	Anti-Angiopoietin 2 antibody (ab56301)	Tris-EDTA buffer (pH 9)	1:200

Transforming growth factor beta 1, TGF- β1; hypoxia inducible factor 1 alpha, HIF-1α; angiopoietin 2, ANGPT-2; ethylene diamine tetra acetate, EDTA.

Hierarchical phase-contrast tomography (HiP-CT) were performed on the beamline BM05 of the European Synchrotron Radiation Facility (ESRF) in Grenoble, France using two different optics covering pixel sizes from 25·5 µm to 6·5 µm and from 6·2 µm to 1·4 µm, respectively. Both of them were mounted with PCO edge 4·2 CLHS cameras. Two acquisition modes were used depending on the size of the organ samples – half and quarter acquisitions. Most scans were performed in half-acquisition mode with the center of rotation on the right side of the field of view (move by typically 900 pixels) to obtain a field of view of 3800 pixels with 6000 projections. For the largest organs, the complete scans at 25 µm were performed using a quarter acquisition protocol based on two scans (one half-acquisition + one annular scan) of 9990 projections each. Once concatenated, the reconstructed field of view is 6000 pixels. In order to cover complete organs or to scan large columns in local tomography, scans were performed with automatic series along the z-axis. The typical sampling step was 2·2 mm vertically for a corresponding beam size of 2·6 mm (overlap of 18%). Nevertheless, some organs scanned at the beginning of the project have been scanned with larger steps (up to 3·6 mm), and larger overlapping (up to 50%).

After preprocessing, the tomographic reconstruction was performed using the filtered back-projection algorithm, coupled with a single distance phase retrieval,^7^ and with a 2D unsharp mask on the projections, as implemented in the ESRF inhouse software PyHST2.^8^ All the sub-volumes are converted into 16 bits, and vertically concatenated (if this had not been done at the previous stage). The remaining ring artefacts were corrected on the reconstructed slices using an in-house Matlab system derived algorithm.^9^ For the most difficult cases, a final correction of the horizontal stripes was performed on the reconstructed volumes after vertical reslicing. Nevertheless, this step was typically not necessary when the stripes have already been corrected on the concatenated radiographs (which became the default reconstruction protocol). Details on samples, scanning procedures and parameters for scanning and reconstruction as well as data links are provided in supplementary Table E3. The tomographic reconstructions were manually segmented for secondary pulmonary lobules by two independent radiologists.^10^ Individual tissue blocks were harvested from those segmented secondary pulmonary lobules. After standard processing, inflammation and fibrotic remodelling were scored semi-quantitatively with regard to their site of manifestation by two independent pulmonary pathologists.

Pairs of secondary pulmonary lobules were manually segmented in Amira v2019·6. Radiomics analysis for each lobule was performed with fixed bin widths of 25, extracting 86 features in total (pyradiomicsV3.0).^11^ Features confounded by volume or duplicated with normalisation, were removed from the feature space. Hierarchical clustering was performed with correlation coefficient cut-off of 0·8. 15 feature groups were found from each group and one parameter with the greatest variance across the six lobules was selected. This reduced feature set was hierarchically clustered to visualise lobule relationships.

For micro-CT analysis, lung biopsies were fixed in 6% formaldehyde and dehydrated using increasing ethanol gradients (30-70-80-90-100%) and 2 hours of hexamethyldisilazane to improve the contrast, followed by air drying. Samples were fixed on a sample holder and scanned using a Skyscan 1272 (Bruker, Kontich, Belgium) using the following settings: 200 mA, 45 kV, 0·2° rotation angle and 3 frames per rotation angle (scan time 45 minutes). Reconstruction of the raw images was performed using NRecon software (Bruker, Kontich, Belgium). 3D reconstructions of the bronchovascular bundle in lungs were performed by region growing using ITK-SNAP 3·8·0 software.^12^

Vascular corrosion casting and scanning electron microscopy was performed using lung tissue of all groups. Briefly, at the time of tissue collection, the afferent vessels were cannulated with an olive-tipped cannula. The vasculature was flushed with saline (at body temperature) followed by glutaraldehyde fixation solution (2·5%, pH 7·4, Sigma Aldrich, Munich, Germany). Fixation was followed by injection of pre-polymerized PU4ii resin (VasQtec, Zurich, Switzerland) mixed with a hardener (40% solvent) and blue dye as casting medium.^13^ After curing of the resin, the lung tissue was macerated in 10% KOH (Fluka, Neu-Ulm, Germany) at 40°C for 2 to 3 days. Specimens were then rinsed with water and frozen in distilled water. The casts were freeze-dried and sputtered with gold in an argon atmosphere and examined using a Philips ESEM XL-30 scanning electron microscope at 15 keV and 21 µA (Philips, Eindhoven, Netherlands).

For quantitative analyses, stereo-pairs with a tilt angle of 6° were collected from each lung explant using a eucentric specimen holder. The stereo-pairs were used for morphometry of parameters defining the architecture of the microvascular unit. The stereo pairs were color-coded and reconstructed as anaglyphic images. With the known tilt angle, calculations were carried out using macros defined by the KS 300 software (Kontron Electronics, Eching, Germany). Intussusceptive angiogenesis was identified via the occurrence of tiny holes with a diameter of 2-5 µm within the casts of capillaries. The quantification of these features was expressed as numerical density per vessel area.^6^^,^^14^, ^15^, ^16^

The microvascular corrosion casts were scanned using Synchrotron radiation tomographic microscopy of corrosion casts (SRXTM) at an X-ray wavelength of 1Å (corresponding to an energy of 12·4 keV) at the Diamond Light Source I13L (Harwell Science and Innovation Campus, Didcot, Oxfordshire, UK). The monochromatic X-ray beam (ΔE/E  =  0·014%) was tailored by a slits system to a profile of 1·4 mm^2^. After penetration of the sample, X-rays were converted into visible light by a thin Ce-doped YAG scintillator screen (Crismatec Saint-Gobain, Nemours, France). Projection images were further magnified by diffraction-limited microscope optics and finally digitalized by a high-resolution CCD camera (Photonic Science, East Sussex, UK). For the tissue samples, the optical magnification was set to 20 ×, and vascular casts were scanned without binning with an optical magnification, resulting in a voxel size of 0·325 μm^3^. For each measurement, 1001 projections were acquired along with dark and periodic flat field images at an integration time of 4 seconds each without binning. Data were post-processed and re-arranged into flat field-corrected sinograms online. Reconstruction of the volume of interest was performed on a 16-node Linux PC Farm (Pentium 4, 2·8 GHz, 512 megabytes RAM) using highly optimized filtered back-projection. A global thresholding approach was used for surface rendering. For 3D visualization and surface rendering, Amira software (Burlington, MA, USA) was installed on an Athlon 64 3500-based computer. The 3D visualizations were analyzed with the program Avizo Fire 7·0.

For the analysis of gene expression and biological pathways, RNA was isolated from n = 15 COVID-19 lung samples, n = 7 influenza A lung samples, n = 18 ILD lung samples (UIP, n = 6; NSIP, n = 6; AFE, n = 6), and n = 25 healthy control lung samples (tissue of one healthy control was accidentally sampled twice) using the Maxwell® RNA extraction system (Promega, Madison, Wisconsin) and following quality control via Qubit analysis (ThermoFisher, Waltham, Massachusetts). All mRNA expression data was obtained via the nCounter® Analysis System (Nanostring Technologies, Seattle, WA) using the PanCancer Progression Panel (770 genes including 30 reference genes). Normalization of raw counts was performed using the nSolver™ analysis software version 3·0 (NanoString Technologies, Seattle, WA) and a modified version of the nCounter® advanced analysis module (version 1·1·5). The normalization process included positive normalization (geometric mean), negative normalization (arithmetic mean) and reference normalization (geometric mean) using the 5 most suitable reference genes from the total of 30 available reference genes selected by the geNorm algorithm.^17^

In order to gain insight on the regulation of physiological functions conveyed by the mRNA expression in COVID-19 and different ILD entities, we made use of the Ingenuity Pathway Analysis (IPA) tool (Qiagen Inc., Venlo, Netherlands). The log_2_ gene expression data of each sample relative to the median gene expression in the controls was committed to the software as an individual observation. The subsequent molecule function activity prediction of IPA was executed with the default settings, thus using information from both in vivo and in vitro experiments. Any z-score obtained as a result gives a quantitative estimate of how a biological function is presumably regulated based on the observed differences in gene expression.^18^ We considered p-values significant according to the following levels of confidence: p < 0·05 (*), p < 0·01 (**) and p < 0·001 (***) as previously described.^15^ Detailed data on gene expression and biological pathways is deposited in Tables E4-E6.

Plasma GDF15, CD163, CXCL12, and MMP1 levels were measured according to the manufacturer's instructions using DuoSet enzyme linked immunosorbent assay (ELISA) from R&D systems (DY957, DY1607, DY1607 & DY350). Assay linearity allowed reliable measurements between 62·5 to 1000 pg/ml for GDF15, 325 to 20,000 pg/ml for CD163, 125 to 2000 pg/ml for CXCL12, and 60 to 4000 pg/ml for MMP1, respectively. Normal plasma levels were confirmed with samples of our own plasma repository, and inter- as well as intra-assay variabilities below 15% were reached using 5 normal and 5 pathological plasmas in 6 replicates. Plasma matricellular markers thrombospondin-2 (TSP2) and insulin like growth factor binding protein 7 (IGFBP7) were measured using in-house developed ELISA. Briefly, 96-well-plates were coated with 100 ng/well of proprietary monoclonal antibodies developed using hybridoma technology. Plates were incubated overnight at 4°C followed by washing with PBS/0·1% Tween-20 and blocking with 1% BSA for 1 h at room temperature. After a second wash, samples along with standards (recombinant TSP2 and IGFBP7 as well as blank controls) were added to the wells, incubated for 2 h at room temperature followed by washing. Goat polyclonal anti-TSP2 and anti-IGFBP7 antibodies purchased from R&D systems (AF1635 & AF1334) were labeled with horseradish peroxidase using a horseradish peroxidase conjugation kit (Abcam, ab102890). Predetermined horseradish peroxidase-antibody dilutions were added to the plates for 1 h, followed by washing and addition of 100 µl ready-to-use tetramethyl-benzidine (Thermofisher; Cat: N301) for 15 minutes at room temperature in the dark. The reaction was stopped with 50 µl 1% HCL, and optical density was measured at 450 nm with 570 nm as reference. The linear range was between 162 to 10,000 pg/ml for TSP2 and 325 to 5,000 pg/ml for IGFBP7. Both assays were highly reliable, and intra- and inter-assay variabilities were below 10% and 13%, respectively. Biomarkers of collagen synthesis were assessed in ethylene-diamine-tetra-acetic acid plasma with validated competitive ELISA developed and performed as described in previous publications for type III collagen formation (PRO-C3),^19^ type IV collagen formation (PRO-C4),^20^ type V collagen formation (PRO-C5),^21^ and type VI collagen formation and endotrophin (PRO-C6)^22^ by Nordic Bioscience (Herlev, Denmark). All samples were measured in duplicates.

Matrix-assisted laser desorption ionization (MALDI)-based mass spectrometry imaging was performed in n = 8 COVID-19 and n = 8 ILD FFPE blocks, as previously described, in order to provide data on metabolic processes in COVID-19 and control fibrotic tissues.^23^, ^24^, ^25^ In brief, FFPE sections (4 μm) were mounted onto indium-tin-oxide-coated glass slides (Bruker Daltonik, Bremen, Germany). The air-dried tissue sections were spray-coated with 10 mg/mL of 9-aminoacridine hydrochloride monohydrate matrix (Sigma–Aldrich, Munich, Germany) in 70% methanol using the SunCollect™ sprayer (Sunchrom, Friedrichsdorf, Germany). Prior to matrix application, FFPE tissue sections were incubated for 1 h at 70°C and de-paraffinized in xylene (2 × 8 min). Spray-coating of the matrix was conducted in 8 passes (ascending flow rates 10 μL/min, 20 μL/min, and 30 μL/min for layers 1–3 and for layers 4–8 with 40 μL/min), utilizing 2 mm line distance and a spray velocity of 900 mm/min. Metabolites were detected in negative ion mode on a 7 T Solarix XR FTICR mass spectrometer (Bruker Daltonik) equipped with a dual electrospray ionization-MALDI source and a SmartBeam-II Nd: YAG (355 nm) laser. Mass spectra were acquired within m/z 75–1,000 with a lateral resolution of 60 µm. L-Arginine was used for external calibration in the electrospray ionization mode. The SCiLS lab software 2020b was used to export the picked peaks of the mass spectra as processed and root mean square normalized imzML files using 4,295 intervals. Annotations were performed using KEGG, where M-H, M-H2O-H, and M+Cl as negative adducts with a mass tolerance of 10 ppm were preferred. We excluded isotopes as well as all irrelevant compounds (drugs, pesticides, etc.). The SPACiAL workflow was used as previously described to annotate connective tissue automatically by means of Masson's trichrome staining.^26^ In short, after MALDI-mass spectrometry imaging analysis, the 9-aminoacridine matrix was removed with 70% ethanol for 5 min from tissue sections, followed by Masson's trichrome (Abcam, # ab150686) staining of the very same tissue sections. The staining was performed according to the manufacturer's instructions. Stained tissue sections were scanned using an AxioScan.Z1 digital slide scanner (Zeiss, Jena, Germany) equipped with a 20x magnification objective. Connective tissue regions were annotated via readout of the blue color channel, and fibrotic regions were selected while manually excluding larger vessels.

Biological networks were analyzed and visualized with Cytoscape (v. 3·8·0). The spatial correlation networks show the absolute value of the correlation coefficient as edges between metabolites/nodes. Compounds with at least one significant correlation are shown. Gene-metabolite networks were created using MetaboAnalyst 5·0 with the complete list of gene identifiers and Kyoto Encyclopedia of Genes and Genomes identifiers as input. The resulting networks were downloaded and further processed in Cytoscape to include information about significantly changed genes and metabolites between the two patient groups.

### Statistics

In this study, the maximum available number of COVID-19 autopsy samples was analyzed. The inclusion criterion was death from COVID-19 but not from life-threatening comorbidities. All analyses were performed unblinded. Statistical analysis was carried out using MedCalc (Ostend, Belgium), GraphPad Prism version 7 (GraphPad Software, Inc., CA, USA), and JMP V8·0·2·2 (SAS Institute Inc. Medmenham Marlow, Buckinghamshire, UK). Baseline characteristics are presented as mean ± standard deviation for continuous variables and as number (frequency) or percentage for categorical variables. To test for normality we used the Shapiro-Wilks test with an alpha of ≤0·05. Statistical analysis of baseline characteristics was performed using Fisher's exact test or Pearson's chi-square test as appropriate for categorical variables as well as paired t-tests or Wilcoxon tests as appropriate for continuous variables. In tissue samples, differences between groups at baseline were assessed using Pearson's chi-square for categorical variables, and analysis of variance (ANOVA, parametric) or Kruskal–Wallis test (nonparametric) for continuous variables.

### Role of funders

Although this study was made possible by a number of funders, the funders had no role in study design, data collection, data analyses, interpretation, trial design, patient recruitment, or writing of this manuscript.

## Results

We analyzed pulmonary autopsy specimens from 31 patients who succumbed to progressive respiratory failure due to severe COVID-19 (mean age 73 ± 11·2 years; 22 male and 9 female subjects; mean hospitalization time 15·3 ± 10·3 days) and compared them to pulmonary autopsy specimens from 7 patients who died from pneumonia caused by influenza A virus subtype H1N1 (mean age 57·4 ± 9·7 years; 5 male and 2 female victims; mean hospitalization time 11·6 ± 5·4 days) and to lung explants of 18 patients with different subtypes of ILD. The ILD group consisted of 6 UIP lungs (0 female and 6 male explants, mean age at transplantation 61·4 ± 6·8 years), 6 NSIP lungs (4 female and 2 male explants, age at transplantation 52·3 ± 9·2 years), and 6 AFE lungs (5 female and 1 male explants, age at transplantation 40·7 ± 27·8 years). AFE lungs included chronic graft-versus-host disease, post-radio/chemotherapy, chronic lung allograft dysfunction and idiopathic pleuro-parenchymatous fibroelastosis all displaying alveolar fibroelastosis as the dominant pattern in histology. Healthy lung tissues from 24 surgically downsized or rejected lung transplants as well as archived non-infected, non-tumor FFPE tissue were used as uninfected control specimens (9 female and 15 male donors, age at specimen retrieval 54·1 ± 24·9 years).On most patients, mRNA expression, plasma, and imaging studies were performed; in a subset of patients also histopathology, micro-CT as well as metabolomics analysis were carried out. With regard to the clinical characteristics of included cases, there was a number of differences between COVID-19 subjects and the other groups: Body mass index was significantly lower in COVID-19 compared to AFE (p = 0·0318 [Fisher's exact test, two-sided]) and age was significantly higher in COVID-19 compared to Influenza A (p = 0·0157 [Fisher's exact test, two-sided]), NSIP (p = 0·0057 [Fisher's exact test, two-sided]), AFE (p = 0·0215 [Fisher's exact test, two-sided]), and healthy controls (p = 0·0054 [Fisher's exact test, two-sided]). Concerning the comorbidities, the proportion of subjects that suffered from hypertension was significantly higher in COVID-19 compared to UIP (p = 0·017 [Fisher's exact test, two-sided]), NSIP (p = 0·017 [Fisher's exact test, two-sided]), and and healthy controls (p = 0·033 [Fisher's exact test, two-sided]). However, the proportion of subjects who received immunosuppressive therapy was significantly lower in COVID-19 compared to UIP (p = 0·004 [Fisher's exact test, two-sided]), NSIP (p < 0·0001 [Fisher's exact test, two-sided]), and AFE (p = 0·022 [Fisher's exact test, two-sided]).

**Figure 2:**
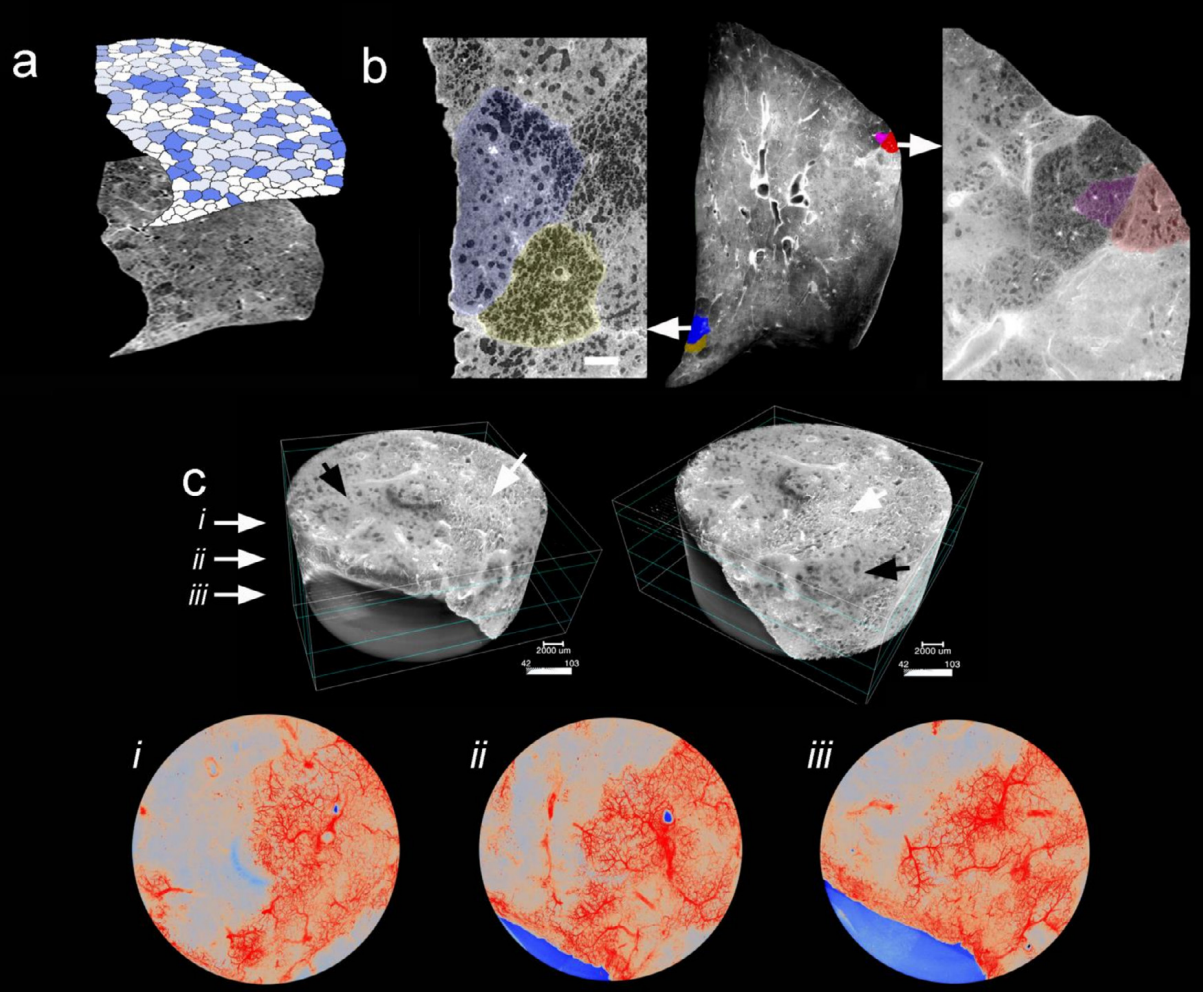
Synchrotron imaging of COVID-19 autopsy lungs. (a) Hierarchical phase-contrast tomography (HiP-CT) reconstructions segmented for secondary pulmonary lobules involved with microischemia. (b) Segmentation of individual secondary pulmonary lobules via HiP-CT depicts the presence of severely abnormal-appearing secondary lobules immediately juxtaposed to nearly normal-appearing lobules. (c) HiP-CT demonstrates the dramatic paucity of blood flow in diseased secondary lobules in the vicinity of fibrotic remodelled areas.

**Figure 3:**
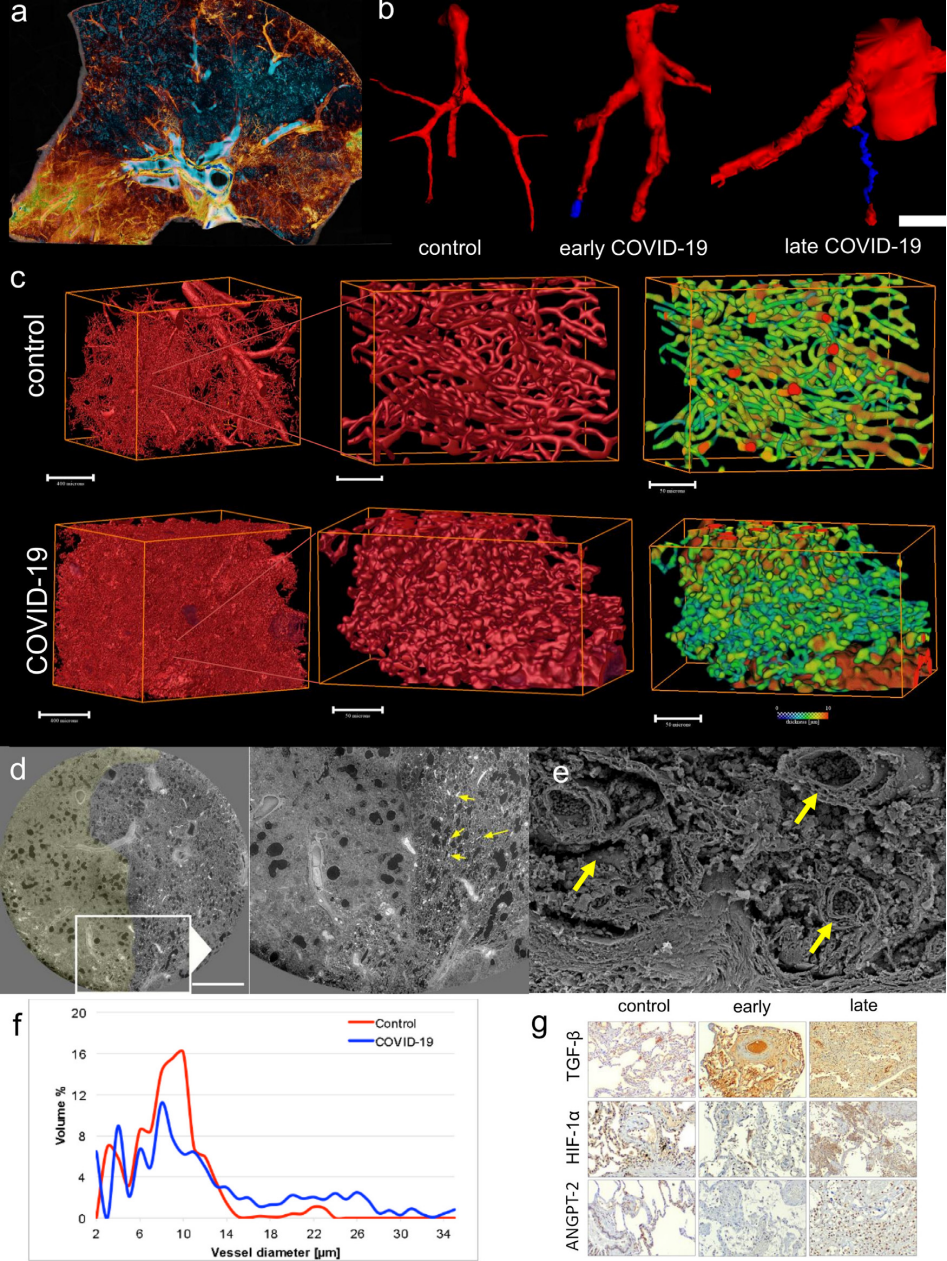
Microvascular alterations in COVID-19 lungs. (a) Volume rendering of a representative hierarchical phase contrast tomography (HiP-CT) slice demonstrates the spatial involvement of microischemia and fibrotic remodelling of airways. Blue color shows regular-shaped airways, whereas yellow depicts the subpleural hot spots of condensation and fibrotic remodelling. (b) Micro-CT-based 3D reconstruction of subsegmental pulmonary arteries (red) and airways (blue) demonstrated (sub-)total occlusion of the arteries in COVID-19-lungs of early and late hospitalized patients as compared to uninfected controls (scale bar equals 200 µm). (c) Three-dimensional evaluation of microvascular corrosion casts by Synchrotron radiation tomographic microscopy illustrating the altered and increased alveolar vascularity in COVID-19 lungs. (d) Representative slices of high-resolution scans from a HiP-CT image of a lung lobe affected by COVID-19 reveals the margin of well-demarcated secondary pulmonary lobules; left with signs of early fibrotic remodelling and microischemia and right with mostly preserved lung parenchyma and increased vascularity (arrows) at the vicinity of the lobular septum. (e) Scanning electron micrograph depicts the dilated blood vessels (arrows) and fibrotic strands along this lobular septum. (f) Volumetric assessment of vessel calibers based on Synchrotron radiation tomographic microscopy of microvascular corrosion casts underlines the occurrence of increased vessel diameters in COVID-19 lungs. (g) Immunohistochemical detection of transforming growth factor beta 1 (TGF-β1), hypoxia inducible factor 1 alpha (HIF1-α), and angiopoietin 2 (ANGPT-2) expression in COVID-19 lungs in the phase of early and late hospitalization compared to controls. Staining results demonstrate the spatial heterogeneity of markers of microischemia. Scale bars equal 600 µm.

Consistent with other reports,^27^^,^^28^ ante-mortem chest computed tomography imaging of the COVID-19 intensive care unit population demonstrated a characteristic patchy or mosaic appearance (Figure 1b). The degree of parenchymal involvement correlated with disease severity and length of hospitalization. To investigate the parenchymal changes associated with the radiographic opacities, we performed high-resolution Synchrotron imaging of the COVID-19 autopsy lungs.^29^^,^^30^ The COVID-19 autopsy lungs were imaged with polychromatic X-ray beam on the beamline BM05 at the European Synchrotron Radiation Facility (ESRF, Grenoble, France) using an average detected energy of 74 keV. The entire organ was imaged at 26·38 µm, zooming locally down to 2·5 µm voxel resolution.^29^ Apart from confirming the common patchy or mosaic appearance of COVID-19 radiographs, Synchrotron imaging demonstrated intralobular septal bands surrounding the patchy areas of disease. The radiographic parenchymal opacities spatially correlated with the distinct anatomical structure referred to secondary lobules (Figure 1c/d and Figure E2). Segmentation of the microcirculation in Synchrotron images revealed a paucity of blood flow in diseased secondary lobules (Figure 2). Comprehensive videos of the Synchrotron-based imaging are deposited in the online supplement (videos E1-E6). In line with a paucity of blood-flow in Synchrotron imaging, micro-CT-based three-dimensional reconstruction of subsegmental pulmonary arteries and airways demonstrated a (sub-) total occlusion of the pulmonary arteries in COVID-19-lungs of early and late hospitalized patients, as compared to uninfected controls (Figure 3). Additionally, scanning electron microscopy of microvascular corrosion casts demonstrated irregular vascular lumens with numerous thromboses and evidence of endothelialitis. In addition, intussusceptive angiogenesis was enhanced in both short- and long-term hospitalizations while sprouting angiogenesis was gradually decreased (Figure E3a and Figure E4).

**Figure 4:**
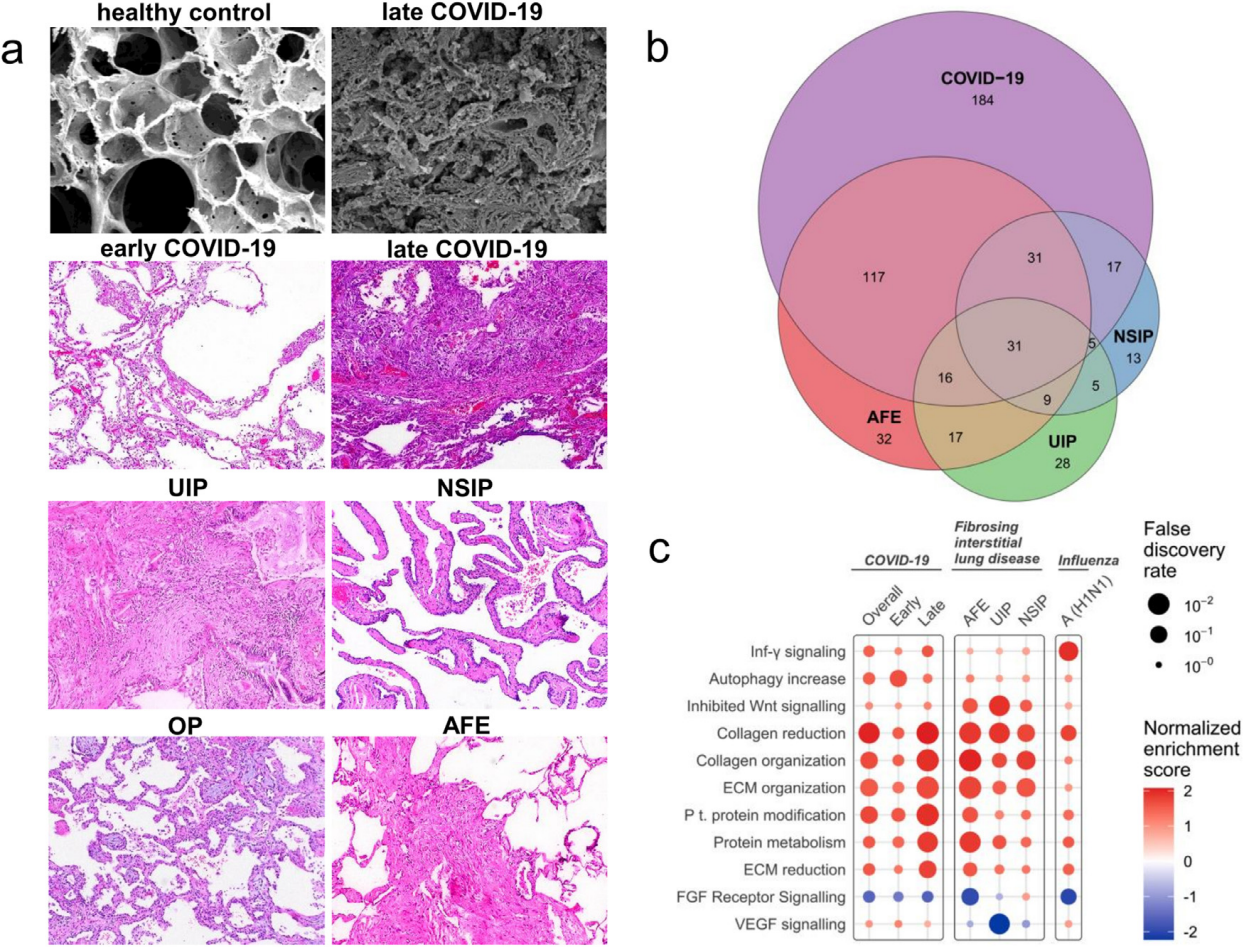
Comparison of COVID-19 with common forms of fibrosing interstitial lung disease (ILD). (a) Different morphological patterns in lungs of early and late hospitalized COVID-19 patients compared to lungs of patients with ILD [usual interstitial pneumonia (UIP), non-specific interstitial pneumonia (NSIP), organizing pneumonia (OP), and alveolar fibroelastosis (AFE) lungs]. Scanning electron microscopy was performed using a Philips XL30 microscope (Philips, Eindhoven, the Netherlands) at 15 keV and 21 μA. Histopathological analysis was performed after hematoxylin and eosin staining. (b) Venn diagram of differentially expressed genes of COVID-19 and influenza lung samples compared with expression in controls (COVID-19, n = 15; influenza A lung samples, n = 7; ILD lung samples, n = 18 (UIP, n = 6; NSIP, n = 6; AFE, n = 6); healthy control lung samples, n = 25). (c) Functional pathway analysis highlights the differential functional gene expression in lung tissue of patients with COVID-19, ILD, and influenza A. Color indicates up- (red) and down (blue)-regulation; Circle size depicts the false discovery rate (FDR). Only significantly up- or down-regulated pathways are shown.

In the complementary histological assessment, diffuse alveolar damage was the predominant pattern in the lungs of COVID-19 patients with short hospitalization time. In line with a paucity of blood-flow in Synchrotron imaging the samples showed hyaline membranes, alveolar septal necrosis, and lymphocytic infiltrates, often accompanied by capillary microthrombi. The histomorphology in these patients was dominated by progressive fibrotic remodelling with thickened alveolar septae, hyperplastic type-II alveolar cells, and often acute fibrinous and organizing pneumonia. In contrast, organizing changes with interstitial myofibroblastic proliferation, septal collagen deposition, and development of loose alveolar plugs of fibroblastic tissue were the predominant changes in the lungs of COVID-19 patients with longer hospitalization time. Compared to healthy controls, early and late deceased COVID-19 patients showed a spatial heterogeneity of angiopoietin 2 (ANPGT1), hypoxia inducible factor 1 alpha (HIF1α), and transforming growth factor beta (TGF-β) as markers of microischemia in immunohistochemistry of affected lung tissue samples (Figure 3g). By definition, lungs from end-stage ILD patients showed typical patterns of advanced fibrotic remodelling: in UIP often with a patchy distribution and concomitant architectural distortion; in NSIP with a more homogenous pattern and a maintained lung architecture accompanied by extended alveolar septal fibrosis; in organizing pneumonia with focal connective tissue plugs within the alveoli and bronchioles; and in AFE with collagen-filled alveoli, and with hyperelastosis of remodelled and paucicellular alveolar walls (Figure 4a).^31^, ^32^, ^33^, ^34^ In total, transcriptome analysis revealed a significantly altered expression signature of 401 genes in COVID-19 compared to healthy controls. In comparison with ILD, the largest overlap was found with AFE (n = 195 genes), while the overlap with both UIP and AFE (n = 111 genes, respectively) was considerably lower (Figure 4b).

Biological pathway analysis using the Qiagen Ingenuity pathway analysis (IPA) tool showed a shift from primarily activated inflammation-related pathways to fibrosis-related pathways over time in COVID-19 lungs (Figure E3b and Figure E5). A decrease in transcripts related to acute inflammation, epithelial-to-mesenchymal transition, and hypoxia was accompanied by an increased mRNA expression linked to fibrotic responses with increasing hospitalization time. The level of angiogenesis-related transcripts in COVID-19 lungs was much greater than in influenza A autopsy specimens (Figure E6 and Figure E7). The trajectory of fibrosis- and angiogenesis-related gene expression in COVID-19 lungs is shown in Figure E3c and Figure E5. Significantly altered mRNA expression signatures were found for the angiogenesis-related genes *flt1*, p = 0·0024 [ANOVA], *hgf*, p = 0·036 [ANOVA], and *vegfa*, p = 0·0054 [ANOVA]. Angiogenesis-related gene expression was decreasing in patients with longer hospitalizations, while *hsp90b1* encoding heat shock protein 90B1 was persistently elevated. The observed fibrotic response to COVID-19 was compared to the gene expression pattern in common ILD as shown in Figure 4c. In COVID-19 lungs, key collagen and matricellular transcripts increased with prolonged hospitalization time equal to, or often greater than in influenza A or ILD. The highest relative extracellular matrix gene expression was found for type III procollagen (*col3a1*), followed by type I procollagen subtypes A1 and A2 (*col1a1 and col1a2*) and lysil oxidase (*lox*) (Figure E8). Spatial metabolomics data related to hypoxia, inflammation, and fibrosis in COVID-19 lung samples are shown in Figure E9.

**Figure 5:**
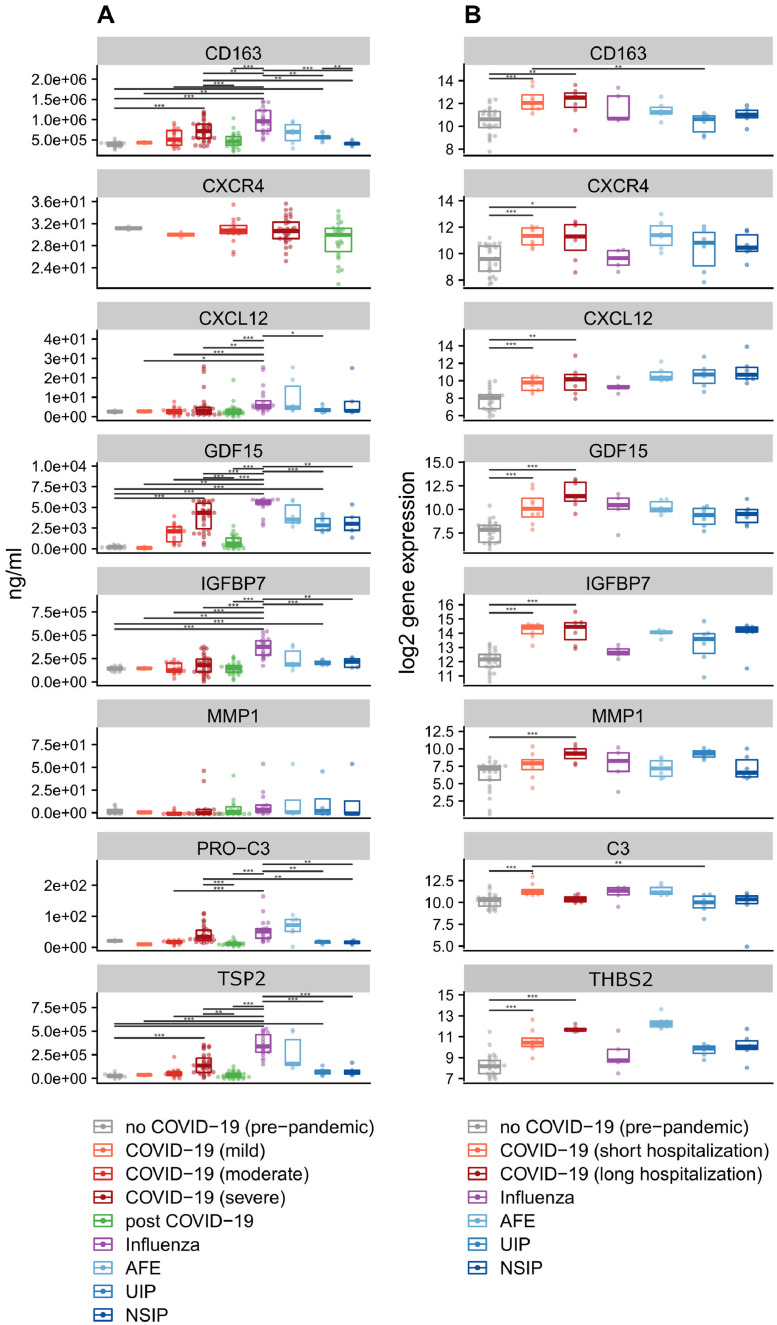
Biomarkers of early fibrotic remodelling, collagen synthesis, and inflammation. (a) Plasma levels of CD163 and the matricellular markers GDF15, MMP1, TSP2, and IGFBP7 were measured in patients with COVID-19, influenza A, and with interstitial lung diseases (UIP, NSIP, and AFE). Mean levels ± standard errors for all time points. Asterisks indicate statistical significance as indicated by bars (*p < 0·05, **p < 0·01, ***p < 0·001). (b) Differential regulation of mRNA expression in lung autopsy tissue of COVID-19 (n = 15) and influenza A (n = 7), and in lung explants from patients with interstitial lung diseases (UIP, n = 6; NSIP, n = 6; AFE, n = 6). Boxplots showing normalized log_2_ counts of mRNA expression ± standard errors of the mean. Asterisks indicate statistical significance as indicated by bars (*p < 0·05, **p < 0·01, ***p < 0·001).

ELISA-based analysis of plasma samples from n = 81 COVID-19 patients, n = 20 influenza A patients, n = 11 ILD patients, and n = 6 ILD patient with acute exacerbation showed increased levels of the fibrogenesis markers thrombospondin 2 (TSP2), glial derived factor 15 (GDF15), insulin like growth factor binding protein 7 (IGFBP7), cluster of differentiation 163 (CD163), and propeptides of procollagen type III, IV and V (PROC3, PROC4, PROC5) in COVID-19 compared to influenza A and ILD entities. Here, TSP2, GFD15, IGFBP7, and to a lesser degree PROC3 and CD163 excellently predicted disease severity. In comparison, *gdf15, igfbp7, thbs2*, and *cd163*, indicators of collagen synthesis and inflammation, showed similar changes towards a pro-fibrotic and pro-collagen synthesis microenvironment during COVID-19 with prolonged hospitalization time in both tissue gene-expression and blood-plasma protein levels. However, compared to conventional ILDs, gene / protein expression in late COVID-19 and post-COVID-19 samples diverged significantly. Notably, the fibrosis markers correlated with their elevated transcript levels in the diseased lungs (Figure 5).

## Discussion

This study comprehensively elucidates the role of microischemia and mosaic involvement of secondary pulmonary lobules employing highest resolution x-ray technology (HiP-CT at the European Synchrotron Radiation Facility in Grenoble, France). We could show that a fatal trajectory of COVID-19 lung disease evolves with a distinct morpho-molecular pattern of fibrotic remodelling, compared to fatal influenza A and end-stage ILD. The techniques employed revealed a unique and prolonged blood vessel neo-formation by intussusceptive angiogenesis preceding fatal fibrotic remodelling in COVID-19 associated with secondary lobular microischemia and consecutive fibrotic pulmonary remodelling. These findings complement our current understanding of the pathophysiology of COVID-19 lung disease, and suggest that treatment strategies aiming at the prevention of microvascular thrombosis and tissue ischemia, such as early oxygen supplementation and judicious anti-coagulation, may have a beneficial effect in patients with severe COVID-19 and prevent subsequent fibrotic remodelling. Moreover, our study shows that biological plasma markers that can be linked to macrophage activation and early fibrotic remodelling, serve as predictors of COVID-19 disease severity and possibly therapeutic response. Further, the herein demonstrated microvascular changes might explain the observed pulmonary symptoms in long-COVID in patients without apparent changes in clinical CT.

Secondary lobules are polygonal-shaped anatomic subunits of the lung supplied by 3 to 5 terminal bronchioles.^10^ Pulmonary arterial vessels are located in the center of each lobule, while vessels of the bronchial circulation are located at the margins of the lobule.^35^ In humans, the secondary lobules are delimited by septal bands—an anatomic feature that facilitates the comparison of neighboring secondary lobules. However, conventional chest computed tomography is not able to delimit these structures. The Synchrotron images demonstrated a high prevalence of fibrotic secondary lobules in the COVID-19 autopsy lungs. Severely abnormal-appearing secondary lobules were frequently juxtaposed to nearly normal-appearing lobules. The observed discrete boundary between secondary lobules suggests an underlying vascular etiology, since an airway-centric inflammation, due to collateral ventilation,^36^ would result in similarly affected adjacent secondary lobules. This hypothesis is further strengthened by the identification of thrombi and microthrombi in Synchrotron image segmentations of the microcirculation causing paucity of blood flow in diseased secondary lobules. Under normal circumstances, localized tissue ischemia within the lung is compensated by the bronchial circulation, which contributes oxygenated blood from the systemic circulation to the ischemic areas. The bronchial vessels branch off the descending aorta to perfuse the proximal airways as well as the fibrous septa of secondary lobules in the lung.^37^ The importance of the bronchial circulation in preventing local tissue ischemia may account for the physical characteristics associated with severe COVID-19; namely, thrombosis of intermediate size vessels of the bronchial circulation.^30^ The loss of this compensatory mechanism may explain a key difference between COVID-19 and other viral infections (e.g., influenza A) and therefore contribute to severe disease courses. These findings suggest that the fibrotic changes found in COVID-19 lungs are the result of lobular microischemia on the level of the secondary pulmonary lobule aggravated by impaired bronchial circulation compensation mechanisms contributing to disease severity.

To further investigate the vascular insufficiency suggested by Synchrotron imaging, we performed corrosion casting of the COVID-19 microcirculation. Consistent with our earlier report,^38^ scanning electron microscopy of microvascular corrosion casts demonstrated microvascular disruption involving irregular vascular lumens with numerous thrombi and evidence of endothelialitis. Consistent with an adaptive response to vascular insufficiency and microischemia, the corrosion casts exhibited a significantly increased intussusceptive angiogenesis in lungs of long- vs short-term hospitalized patients — a hallmark that divides COVID-19 from influenza A and other fibrotic lung diseases. From these data we are able to conclude that the increasing prevalence of intussusceptive angiogenesis with longer hospitalization time likely results from continuing microischemia. Notably, the prominence of intussusceptive angiogenesis, in contrast to sprouting angiogenesis, may reflect its adaptive role in prolonged tissue ischemia and pulmonary fibrogenesis^38^^,^^39^ and may be directly linked to the infection of endothelial cells with SARS-CoV-2 and respective pro-angiogenic changes in gene expression.^40^ The morphologic evidence of intussusceptive angiogenesis was supported by enhanced expression of angiogenesis-associated genes while transcriptome profiling of the COVID-19 lungs identified a progressive decrease in transcripts related to acute inflammation, epithelial-mesenchymal transition, and hypoxic responses, contrasting with an increase in transcripts linked to fibrotic tissue remodelling. Of note, the pro-angiogenic mRNA expression was much greater in COVID-19 lungs compared to influenza A autopsy specimens and stronger in patients who deceased after short rather than longer hospitalization.

Patterns of fibrotic matrix remodelling in response to lobular microischemia were explored in more detail using mRNA expression analysis combined with MALDI mass spectrometry imaging-based tissue metabolomics. The expression of key genes related to extracellular matrix formation was highly increased in COVID-19 lungs, and uniformly equal to, or greater than in severe influenza A or ILD. Moreover, there was a general trend for key collagen and matricellular transcripts to increase with prolonged hospitalization time, in line with previously published data^41^, ^42^, ^43^), identifying *col3a1, col1a1, col1a2*, and *lox* as the most abundant connective tissue molecules in COVID-19. Notably, the highly activated fibrogenesis in severe COVID-19 was also reflected by highly elevated plasma levels of the corresponding fibrogenesis markers.^44^ These results are reflected by the histological trajectory of severe COVID-19 with progressive fibrosing and organizing changes over time, as described above.

The microischemia-induced fibrotic remodelling to COVID-19 was compared to common ILD: UIP, NSIP, and AFE. Although UIP, the most common histologic pattern in fibrosing ILD, is associated with angiogenesis, it typically demonstrates more sprouting than intussusceptive angiogenesis.^45^ Moreover, angiogenesis in UIP is associated with a chaotic tumor-like aberrant distribution of vessels. While NSIP and AFE are linked to enhanced intussusceptive angiogenesis, they both demonstrate a diffuse and more uniform pattern of inflammation than that observed in COVID-19.^45^^,^^15^ Although similarities between the histological presentation and the genetic background of severe COVID-19 and UIP have been described,^46^^,^^47^ our data show that gene expression, plasma protein expression, and disease trajectory in COVID-19 are distinct from the common forms of ILD, including, e.g., key genes related to inflammation (gdf15, cd163), angiogenesis (cxcr4, cxcl12), and fibrosis (mmp1, col3a1), potentially reflecting the unique vascular etiology of COVID-19.

In summary, our data demonstrate that the mosaic radiographic appearance of COVID-19 reflects the secondary lobule—the lung anatomic compartment that is the watershed of the pulmonary and bronchial circulation. Based on our data, we hypothesize that in patients with compromised pulmonary and bronchial circulation, severe COVID-19 causes microischemia of the secondary lobule. Irreversible tissue ischemia induces fibrotic changes in the lung that are associated with long-haul COVID-19 symptoms.

We acknowledge that our study has limitations. Often, there are inherent differences in disease incidence between the different fibrotic lung diseases analyzed in this study regarding age and sex. In addition, ILD samples were obtained from end-stage disease lung explants and a lung transplantation is rarely performed after the age of 60. For this reasons and due to limited sample sizes (n< 10), we were often not able to correct for these confounding factors. For the sake of consistency, we did not perform multivariate analysis for the COVID-19 and Influenza comparisons were this might have been possible. One limitation of our study is the use of autopsy samples. Sometimes, their tissue quality is limited due to prolonged ischemia time before workup and concomitant autolysis which in turn may restrict the validity of gene expression analyses. However, all samples used were selected with the utmost care to provide the best possible tissue quality. One more limitation is the fact that the Influenza A samples date back to the 2009 swine flu pandemic while COVID-19 samples were obtained at least 10 years later. Given that medical treatment refines continuously, this time gap may also potentially confound our findings. Although a major limitation of the present study is the limited number of patients included - which is in turn due the use of autopsy specimens, the central implications of our observations include the potential use of plasma markers to detect early fibrotic remodelling and the use of aggressive respiratory support to minimize tissue ischemia.

## Contributors

MA, JCK, PDL, DS, SJM, and DDJ conceptualized the project and designed experiments; MA, CW, AT, PB, FPL, SEV, TI, MPK, PT, and AJB performed autopsies and sample preparation; PT, CLW, and AJB designed and built instrumentation and performed HiP-CT imaging; SM designed sample holders; PT designed and implemented tomographic reconstruction methods; CLW and WL designed, managed and performed image analysis; JCK, HMK, HHK, AH, TW, MMH, SMB, and SJM provided medical interpretation and regions of interest; MA, JCK, SJM, and DDJ wrote the paper; RS, DS, DJL, and MAK established and determined serum markers of fibrosis and inflammation; EB, CW, HS, CLW, VP, WLW, CD, AJB, MPK, AW, PDL, PT, and DDJ assisted with image and data analysis. BS and SD were active in patient care, obtained, processed, and stored plasma samples and generated the corresponding clinical data. HS and MPK performed the statistical analysis and verified the underlying data of the manuscript from the academic team. All authors assisted in reviewing and revising the manuscript and all authors read and approved the final version of the manuscript. JCK, MA, MMH, DS, SJM, and DDJ were responsible for the decision to submit the manuscript.

## Data sharing statement

Anonymized raw data and the results of all molecular analyses are available for 10 years from publication upon reasonable request to the corresponding author. All data are deposited in an online research data repository (doi: https://doi.org/10.6084/m9.figshare.20749609.v1).

## Declaration of interests

HHK received fees for lectures and consultations from Roche Pharma AG, Novartis, AstraZeneca, Genomic Health, Pfizer, and Amgen, all outside the present study. MMH received fees for lectures and consultations from Acceleron, Actelion, Bayer, GSK, Janssen, MSD, and Pfizer, all outside the present study. TW declares funding by the German Ministry of Research and Education. MAK and DJL declare the possession of “Nordic Bioscience” stock options. BS received fees for lectures from Boehringer Ingelheim. The other authors have no potential conflicts of interest to report.

## Appendix Supplementary materials

Image, application 1

Video E1. Image showing the upper right lung lobe of a 54-year-old male who died of COVID-19. Overview of the lobe imaged at 25 µm/voxel shows the mosaic patterns of damage that coincide with pulmonary lobule boundaries. Zoom in shows two adjacent lobules with strikingly different patterns of disease. Pan out shows two additional pairs of lobules (red and pink, cyan and orange). These pairs were used in radiomics analysis. Image, application 2

Video E2. Three-Dimensional complexity of vascular remodelling and bronchio-pulmonary shunting in COVID-19 highlighted by Hierarchical Phase-Contrast Tomography (HiP-CT). Segmentation into distal bronchiolus (blue) paralleled by a pulmonary artery (red) and an expanded plexus of vasa vasorum (yellow) from a 6·5 µm/voxel volume of interest with the upper right lung lobe of a 54 year old male. Arteriovenous anastomoses (yellow) can be seen between bronchial arteries and pulmonary artery vasa vasorum, which drain into pulmonary veins. The perivascular vasa vasorum and bronchial artery plexus are characterized by complex nodular lesions with angiomatoid- and plexiform-like arrangements. Image, video 3

Video E3. Video showing slices (1mm thick projections) through the upper right lung lobe of a 54 year old male who died of COVID-19 images at 25 um/voxel. Images are filtered with a standard deviation map, which highlight areas of high intensity gradient. Blue areas show the air spaces which are open and have low intensity variation. Red-yellow highlight subpleural hotspots of with high standard deviation which are typically highly vascularized or show extensive condensation and fibrotic remodelling. Image, video 4

Video E4. Slices through the upper left lung lobe of a COVID-19 patient images at 25um/voxel. Slices show the same mosaic type patterning seen in previous COVID-19 lung imaged with HiP-CT. Image, video 5

Video E5. Slices through the upper left lung lobe of a COVID-19 patient; images at 25 µm/voxel. Slices show the same mosaic type patterning seen in previous COVID-19 lung imaged with HiP-CT. Image, video 6

Video E6. Volume of interest imaged at 6.24 µm/voxel within the upper right lung lobe of a 54 year old male who died of COVID-19. The column shows the boundary between two lobules with varying patterns of damage and vascularity. HiP-CT demonstrates the dramatic paucity of blood flow in diseased secondary lobules in the vicinity of fibrotic remodelled areas. Image, video 7

Image, video 8

Image, application 3

Image, application 4

Image, application 5

Image, application 6

Image, application 7

Image, application 8

Image, application 9

Image, application 10

Image, application 11

Image, application 12
