# Supplementary material for: The fatal trajectory of pulmonary COVID-19 is driven by lobular ischemia and fibrotic remodelling
Source: eBioMedicine. 2022 Oct 4;85:104296. doi: 10.1016/j.ebiom.2022.104296 (PMC9535314; doi:10.1016/j.ebiom.2022.104296)
Supplement: Supplementary file 1 [file mmc1.docx]

**Table E1. Clinical characteristics – tissue samples**

| **Disease** | **Number of cases** | **Age (years)** | **Male sex** | **BMI** | **Hospitalization time** | **IS** | **RAAS-IA** | **Hypertension** | **Active smoking** | **DM type II** |
| --- | --- | --- | --- | --- | --- | --- | --- | --- | --- | --- |
|  |  |  |  |  |  |  |  |  |  |  |
| COVID-19 | 16 | 75±10 | 63% | 28±7,9 | 9±5,8 days | 6% (25% n/a) | 44% (31% n/a) | 75% (19% n/a) | 25% (25% n/a) | 44% (6% n/a) |
|  |  |  |  |  |  |  |  |  |  |  |
| Influenza A | 7 | 53±9 | 83% | 35,2±9,7 | 11±5 days | 0% | 43% | 86% | 57% | 0% |
|  |  |  |  |  |  |  |  |  |  |  |
| ILD | 18 | 56±15,6 | 50% | 24,2±4,7 |  | 83% | 11% | 39% | 33% | 6% |
|  |  |  |  |  |  |  |  |  |  |  |
| UIP/IPF | 6 | 61±6,2 | 100% | 28,1±4,2 |  | 83% | 0% | 33% | 50% | 0% |
|  |  |  |  |  |  |  |  |  |  |  |
| NSIP | 6 | 48,5±8,3 | 33% | 24,2±3,2 |  | 100% | 17% | 33% | 33% | 0% |
|  |  |  |  |  |  |  |  |  |  |  |
| AFE | 6 | 52,5±23 | 17% | 21,6±3,4 |  | 83% | 17% | 50% | 17% | 17% |
|  |  |  |  |  |  |  |  |  |  |  |
| Healthy | 23 | 60±25 | 61% | 24±3,7 |  | 4% (52% n/a) | 9% (57% n/a) | 30% (39% n/a) | 17% (9% n/a) | 13% (39% n/a) |
