# Supplementary material for: The fatal trajectory of pulmonary COVID-19 is driven by lobular ischemia and fibrotic remodelling
Source: eBioMedicine. 2022 Oct 4;85:104296. doi: 10.1016/j.ebiom.2022.104296 (PMC9535314; doi:10.1016/j.ebiom.2022.104296)
Supplement: Supplementary file 2 — Video E1. Image showing the upper right lung lobe of a 54-year-old male who died of COVID-19. Overview of the lobe imaged at 25 µm/voxel shows the mosaic patterns of damage that coincide with pulmonary lobule boundaries. Zoom in shows two adjacent lobules with strikingly different patterns of disease. Pan out shows two additional pairs of lobules (red and pink, cyan and orange). These pairs were used in radiomics analysis. [file mmc2.docx]

**Table E2. Clinical characteristics – plasma samples**

| **Disease** | **Number of cases** | **Age (years)** | **Male sex** | **Hospitalization time** | **Active smoking** | **DM type II** | **COPD** | **Cardiac disease** | **Adipositas** |
| --- | --- | --- | --- | --- | --- | --- | --- | --- | --- |
|  |  |  |  |  |  |  |  |  |  |
| COVID-19 | 81 | 54±18 | 67% | 24±35 days | 4% (14% n/a) | 17% (4% n/a) | 11% (7% n/a) | 27% (5% n/a) | 28% (5% n/a) |
| - mild | 4 | 33±25 | 25% | 13±42 days | 0% | 0% | 0% | 25% | 0% |
| - moderate | 16 | 64±19 | 75% | 1415 days | 25% | 31% (6% n/a) | 6% (19% n/a) | 44% (19% n/a) | 25% (19% n/a) |
| - severe | 33 | 55±17 | 30% | 17±18 days | 7% (36% n/a) | 21% (6% n/a) | 12% (9% n/a) | 27% (12% n/a) | 42% (3% n/a) |
| - post-COVID | 28 | 52±15 | 64% | 74±26 days | 0% (4% n/a) | 14% | 14% | 18% | 18% |
|  |  |  |  |  |  |  |  |  |  |
| Influenza A | 20 | 57±12 | 85% |  |  |  |  |  |  |
|  |  |  |  |  |  |  |  |  |  |
| ILD | 17 | 69±12 | 76% |  |  |  |  |  |  |
|  |  |  |  |  |  |  |  |  |  |
| UIP/IPF | 6 | 70±10 | 50% |  |  |  |  |  |  |
|  |  |  |  |  |  |  |  |  |  |
| NSIP | 5 | 77±12 | 100% |  |  |  |  |  |  |
|  |  |  |  |  |  |  |  |  |  |
| AE-ILD | 6 | 59±11 | 83% |  |  |  |  |  |  |
