# Supplementary material for: The fatal trajectory of pulmonary COVID-19 is driven by lobular ischemia and fibrotic remodelling
Source: eBioMedicine. 2022 Oct 4;85:104296. doi: 10.1016/j.ebiom.2022.104296 (PMC9535314; doi:10.1016/j.ebiom.2022.104296)
Supplement: Supplementary file 9 [file mmc9.docx]

**Table E2a.** Statistical comparison of patient characteristics.

| **Tissue samples** | | | | | |
| --- | --- | --- | --- | --- | --- |
|  |  |  |  |  |  |
| **BMI – tissue samples** | **COVID-19** | **Influenza A** | **UIP** | **NSIP** | **AFE** |
| **Influenza A** | 0.4212 |  |  |  |  |
| **UIP** | 0.5258 | 0.4352 |  |  |  |
| **NSIP** | 0.1353 | 0.1712 | 0.0947 |  |  |
| **AFE** | 0.0318 | 0.2274 | 0.0215 | 0.2204 |  |
| **Healthy** | 0.1928 | 0.0865 | 0.4838 | 0.6299 | 0.9486 |
|  |  |  |  |  |  |
| **Age – tissue samples** | **COVID-19** | **Influenza A** | **UIP** | **NSIP** | **AFE** |
| **Influenza A** | 0.0157 |  |  |  |  |
| **UIP** | 0.0532 | 0.9786 |  |  |  |
| **NSIP** | 0.0057 | 0.2195 | 0.0843 |  |  |
| **AFE** | 0.0215 | 0.2709 | 0.3212 | 0.7228 |  |
| **Healthy** | 0.0054 | 0.4408 | 0.0130 | 0.0499 | 0.0684 |
|  |  |  |  |  |  |
| **Sex – tissue samples** | **COVID-19** | **Influenza A** | **UIP** | **NSIP** | **AFE** |
| **Influenza A** | 1 |  |  |  |  |
| **UIP** | 0.133 | 0.462 |  |  |  |
| **NSIP** | 0.348 | 0.286 | 0.061 |  |  |
| **AFE** | 0.149 | 0.103 | 0.015 | 0.505 |  |
| **Healthy** | 1 | 1 | 0.137 | 0.364 | 0.080 |
|  |  |  |  |  |  |
| **Smoking – tissue samples** | **COVID-19** | **Influenza A** | **UIP** | **NSIP** | **AFE** |
| **Influenza A** | 1 |  |  |  |  |
| **UIP** | 1 | 1 |  |  |  |
| **NSIP** | 1 | 1 | 0.558 |  |  |
| **AFE** | 0.6 | 0.559 | 0.545 | 1 |  |
| **Healthy** | 0.23 | 0.318 | 0.29 | 0.588 | 1 |
|  |  |  |  |  |  |
| **Hypertension – tissue samples** | **COVID-19** | **Influenza A** | **UIP** | **NSIP** | **AFE** |
| **Influenza A** | 1 |  |  |  |  |
| **UIP** | 0.017 | 0.103 |  |  |  |
| **NSIP** | 0.017 | 0.103 | 1 |  |  |
| **AFE** | 0.071 | 0.266 | 1 | 0.558 |  |
| **Healthy** | 0.033 | 0.174 | 0.642 | 0.642 | 1 |
|  |  |  |  |  |  |
| **Diabetes type II – tissue samples** | **COVID-19** | **Influenza A** | **UIP** | **NSIP** | **AFE** |
| **Influenza A** | 0.051 |  |  |  |  |
| **UIP** | 0.061 | - |  |  |  |
| **NSIP** | 0.061 | - | - |  |  |
| **AFE** | 0.336 | 0.462 | 1 | 1 |  |
| **Healthy** | 0.245 | 0.521 | 0.521 | 0.521 | 1 |
|  |  |  |  |  |  |
|  |  |  |  |  |  |
| **Immunosuppression – tissue samples** | **COVID-19** | **Influenza A** | **UIP** | **NSIP** | **AFE** |
| **Influenza A** | 1 |  |  |  |  |
| **UIP** | 0.004 | 0.005 |  |  |  |
| **NSIP** | 0 | 0.001 | 1 |  |  |
| **AFE** | 0.022 | 0.021 | 1 | 0.455 |  |
| **Healthy** | 1 |  | 0.005 | 0.001 | 0.028 |
|  |  |  |  |  |  |
| **RAAS-IA-drugs – tissue samples** | **COVID-19** | **Influenza A** | **UIP** | **NSIP** | **AFE** |
| **Influenza A** | 0.63 |  |  |  |  |
| **UIP** | 0.035 | 0.192 |  |  |  |
| **NSIP** | 0.131 | 0.559 | 1 |  |  |
| **AFE** | 0.131 | 0.559 | 1 | 1 |  |
| **Healthy** | 0.08 | 0.593 | 0.5 | 1 | 1 |
|  |  |  |  |  |  |
|  |  |  |  |  |  |
| **Plasma samples** | | | | |  |
|  |  |  |  |  |  |
| **Age – plasma samples** | **COVID-19** | **Influenza A** | **UIP** | **NSIP** |  |
| **Influenza A** | 0.5976 |  |  |  |  |
| **UIP** | 0.0188 | 0.0111 |  |  |  |
| **NSIP** | 0.0211 | 0.0128 | 0.8552 |  |  |
| **ILD-ARDS** | 0.2532 | 0.2782 | 0.1895 | 0.1892 |  |
|  |  |  |  |  |  |
| **Sex – plasma samples** | **COVID-19** | **Influenza A** | **UIP** | **NSIP** |  |
| **Influenza A** | 0.108* |  |  |  |  |
| **UIP** | 0.411 | 0.112 |  |  |  |
| **NSIP** | 0.32 | 1 | 0.182 |  |  |
| **ILD-ARDS** | 0.659 | 1 | 0.545 | 1 |  |
|  |  |  |  |  |  |

Categorical variables were analyzed using Fisher’s exact test unless stated otherwise. Continuous variables were analyzed using paired t-tests unless stated otherwise. *P-values calculated using the chi square test.
