# Supplementary figures and images for: The fatal trajectory of pulmonary COVID-19 is driven by lobular ischemia and fibrotic remodelling
Source: eBioMedicine. 2022 Oct 4;85:104296. doi: 10.1016/j.ebiom.2022.104296 (PMC9535314; doi:10.1016/j.ebiom.2022.104296)

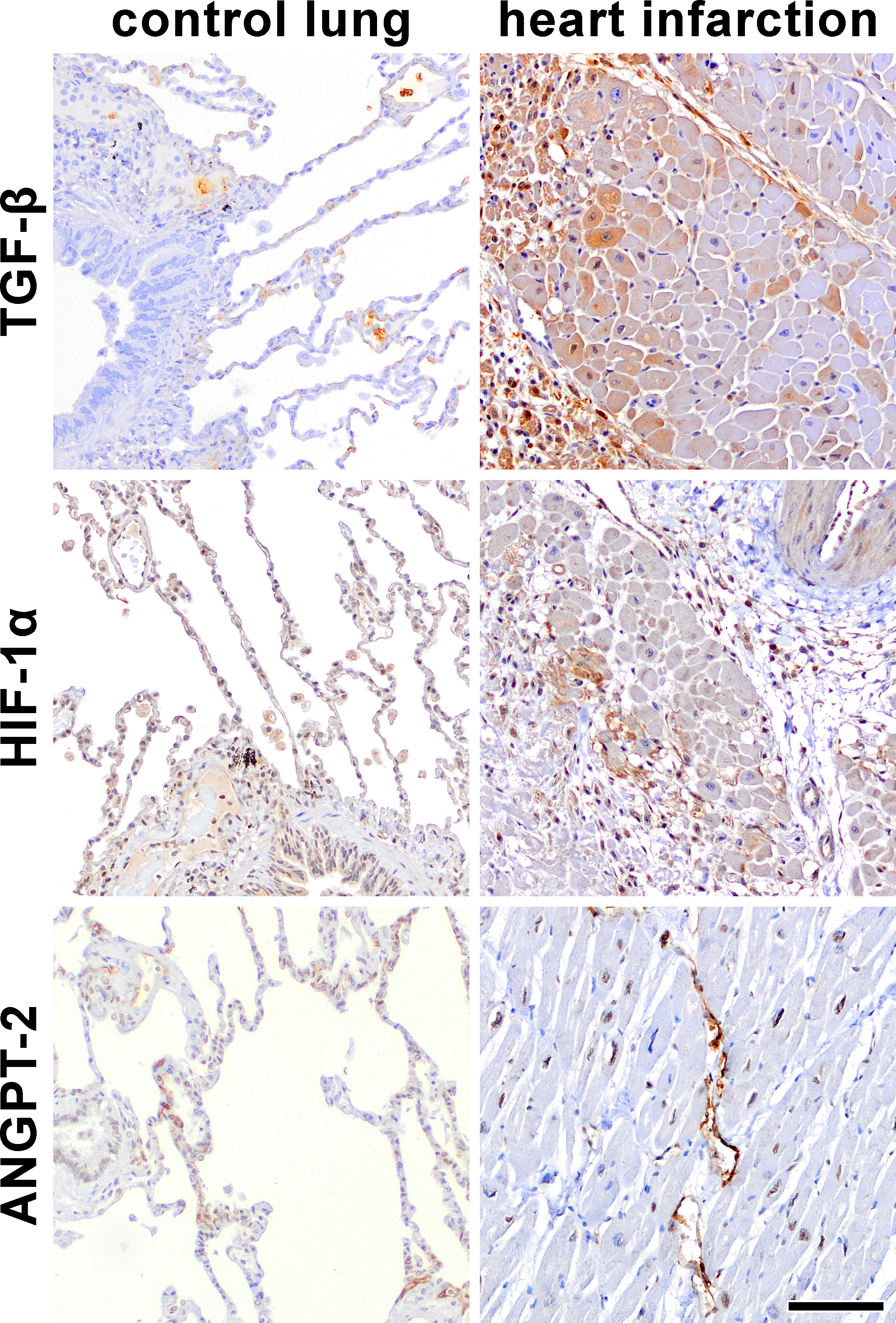

Supplement: Supplementary file 10 [file mmc10.zip › mmc10.tif]

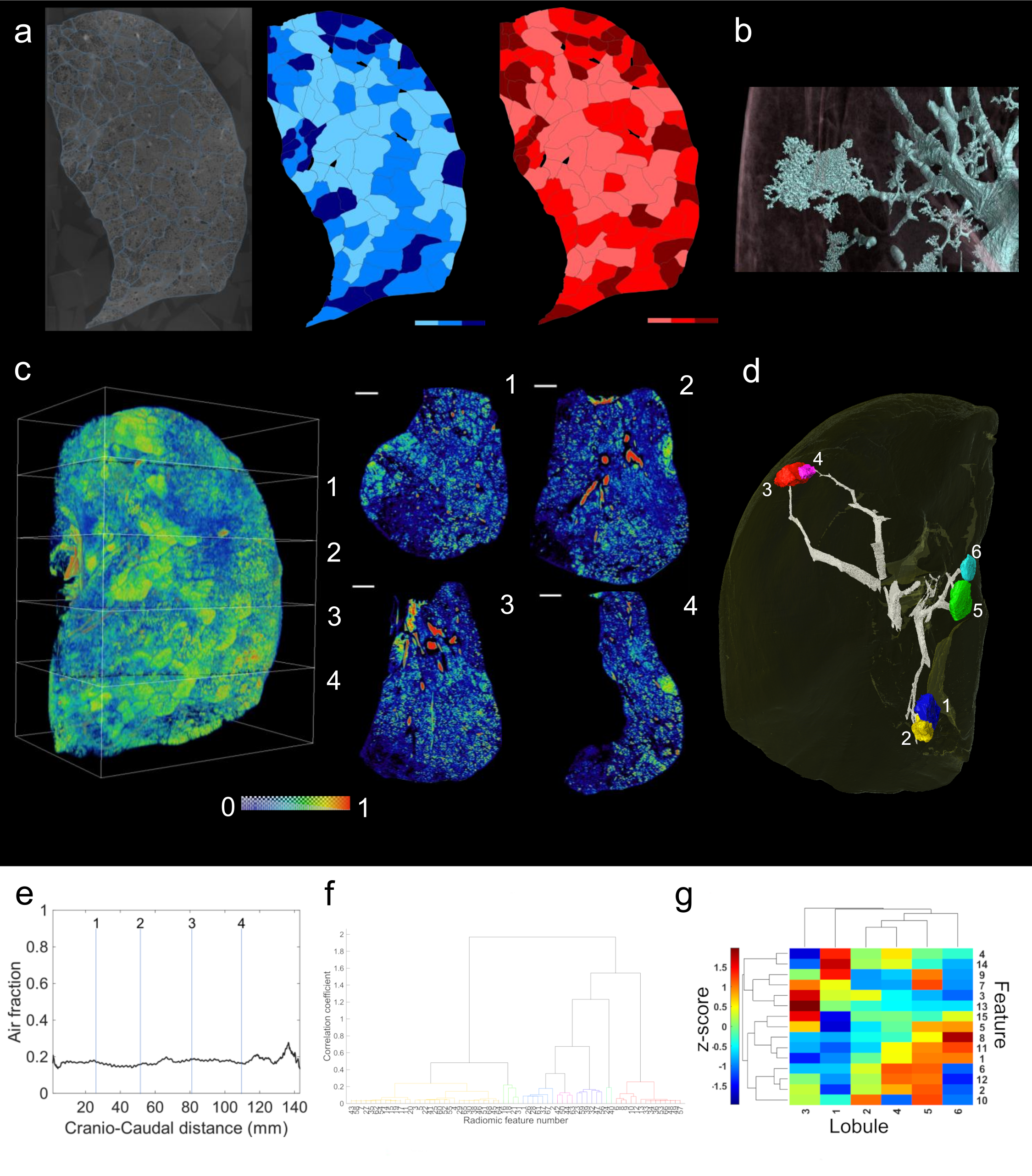

Supplement: Supplementary file 11 [file mmc11.zip › mmc11.tif]

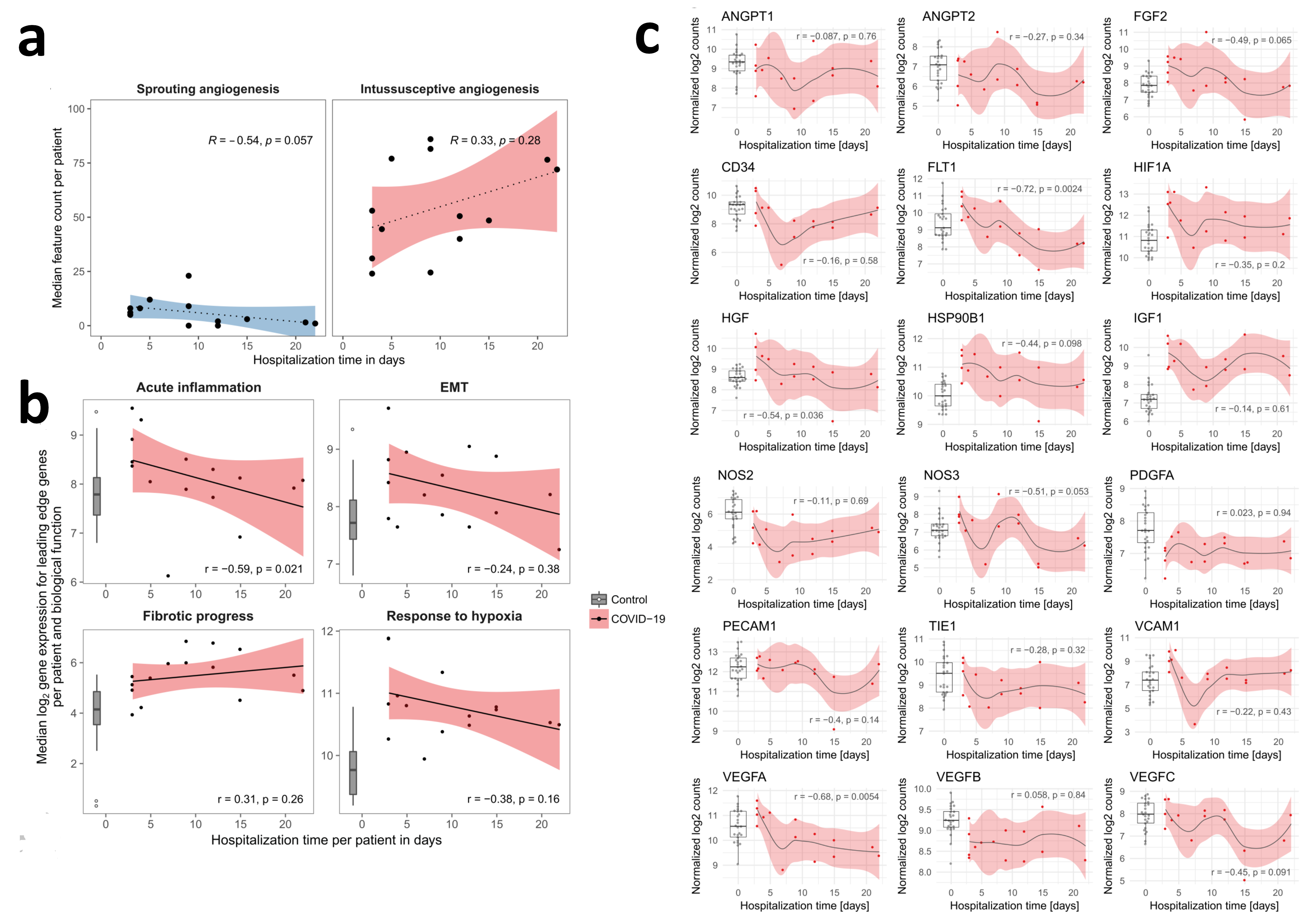

Supplement: Supplementary file 12 [file mmc12.zip › mmc12.tif]

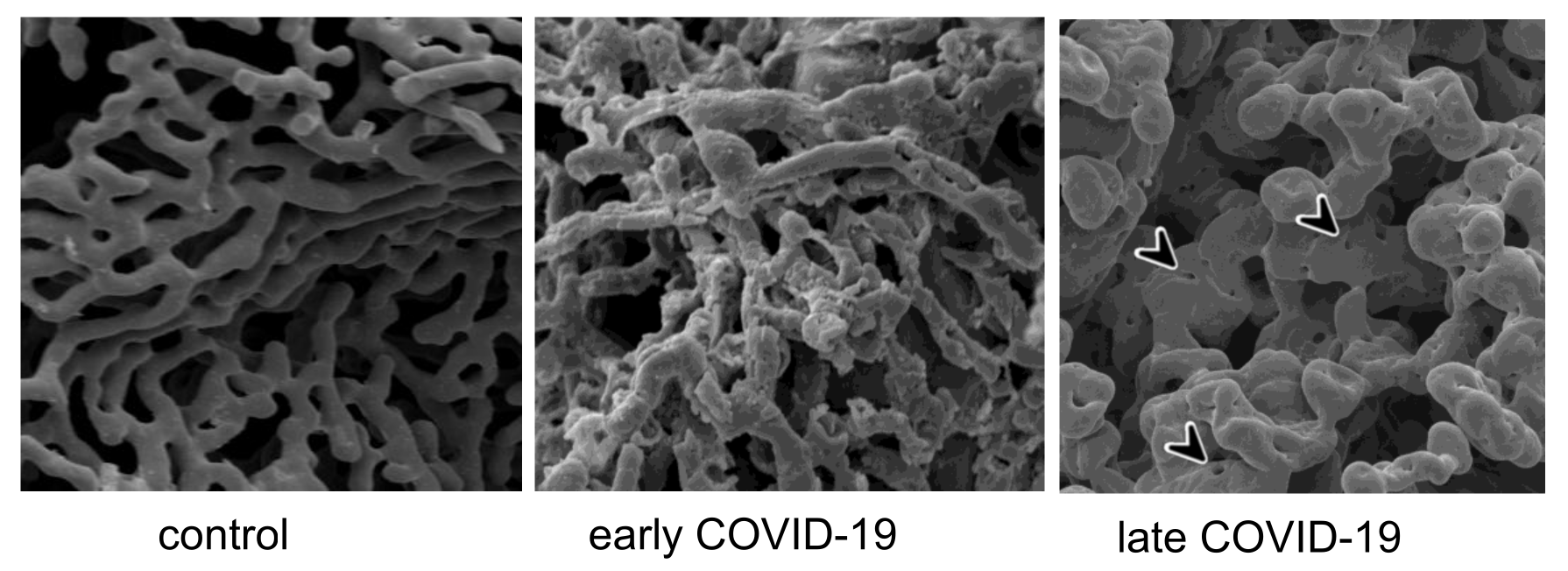

Supplement: Supplementary file 13 [file mmc13.zip › mmc13.tif]

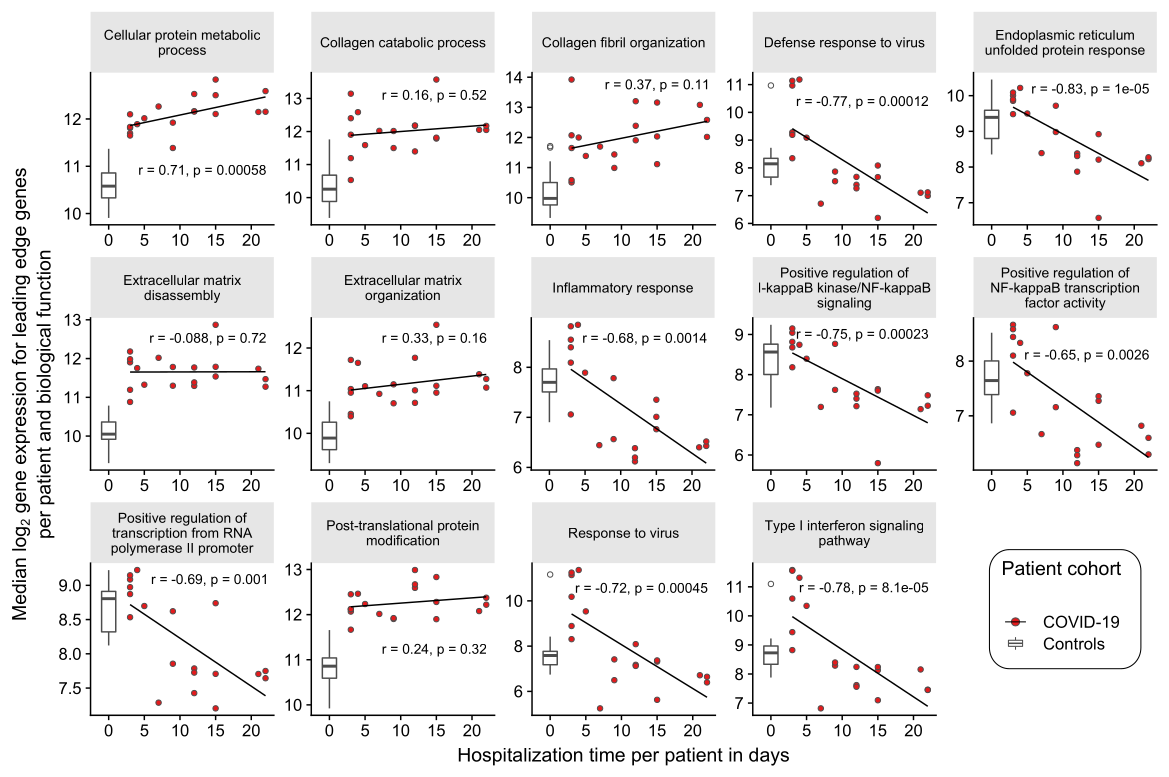

Supplement: Supplementary file 14 [file mmc14.zip › mmc14.tif]

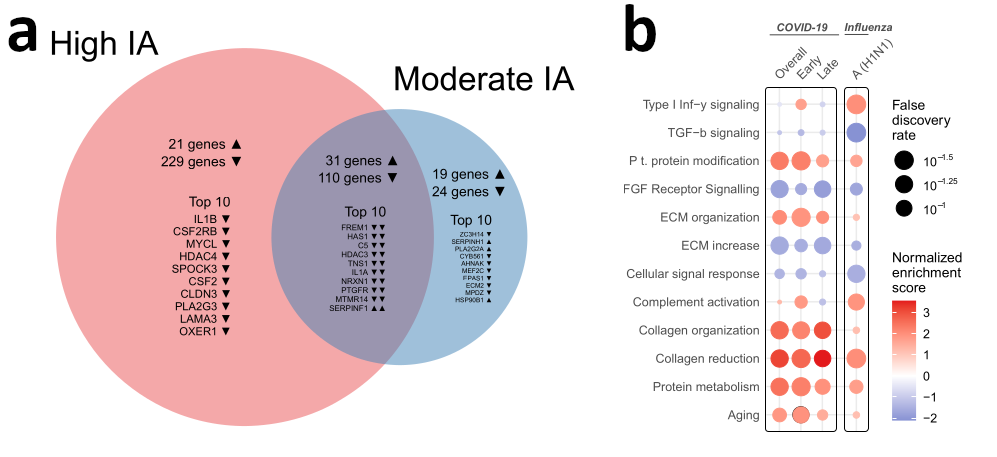

Supplement: Supplementary file 15 [file mmc15.zip › mmc15.tif]

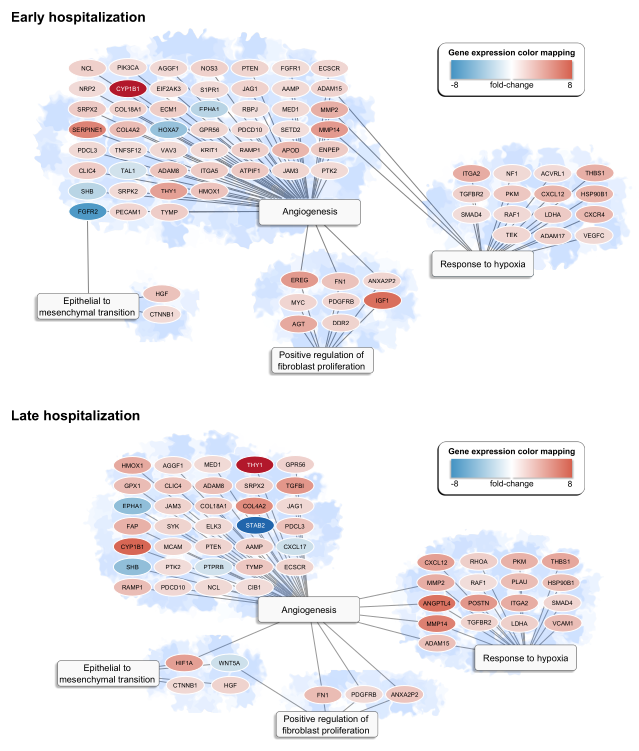

Supplement: Supplementary file 16 [file mmc16.zip › mmc16.tif]

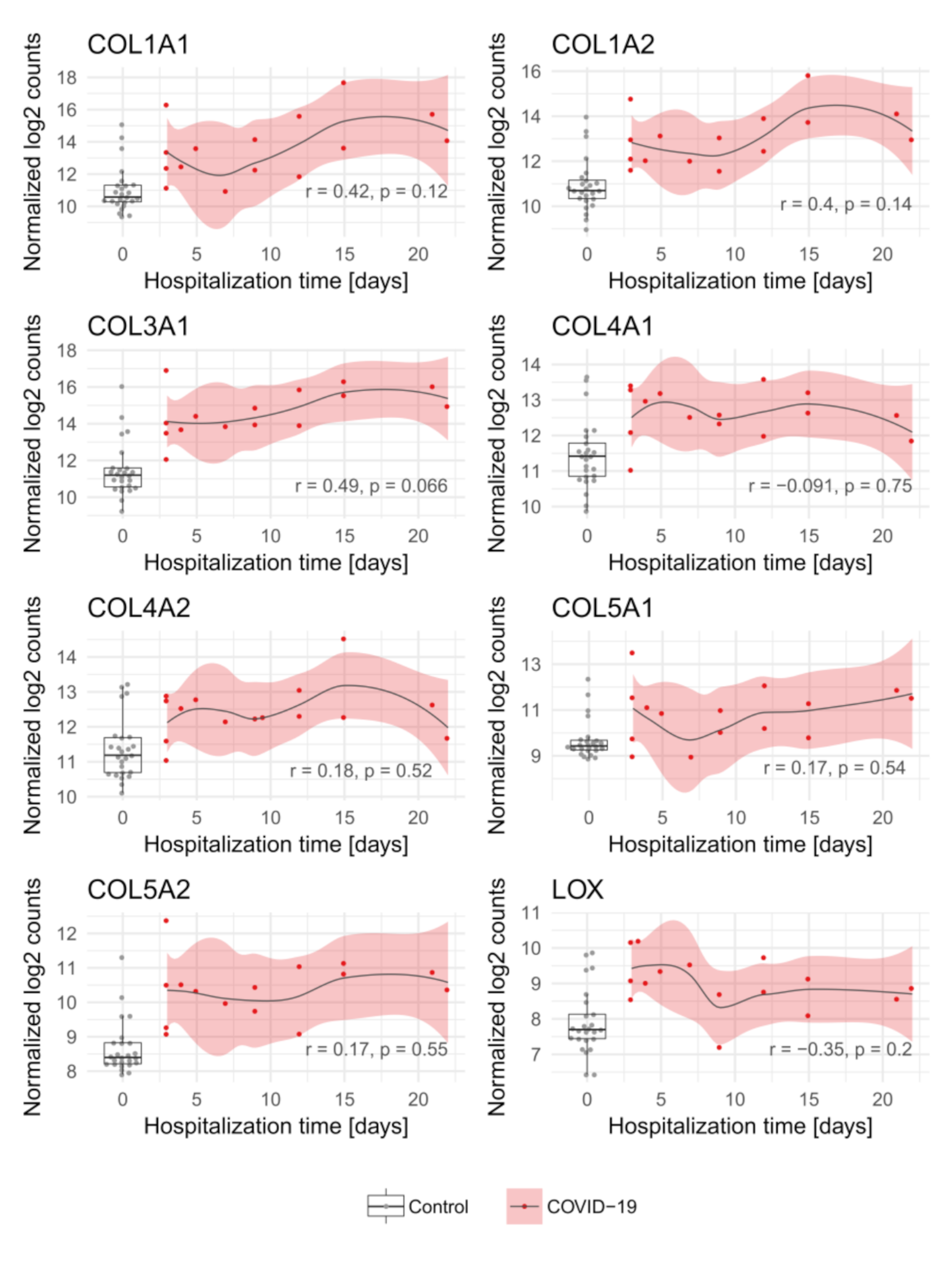

Supplement: Supplementary file 17 [file mmc17.zip › mmc17.tif]

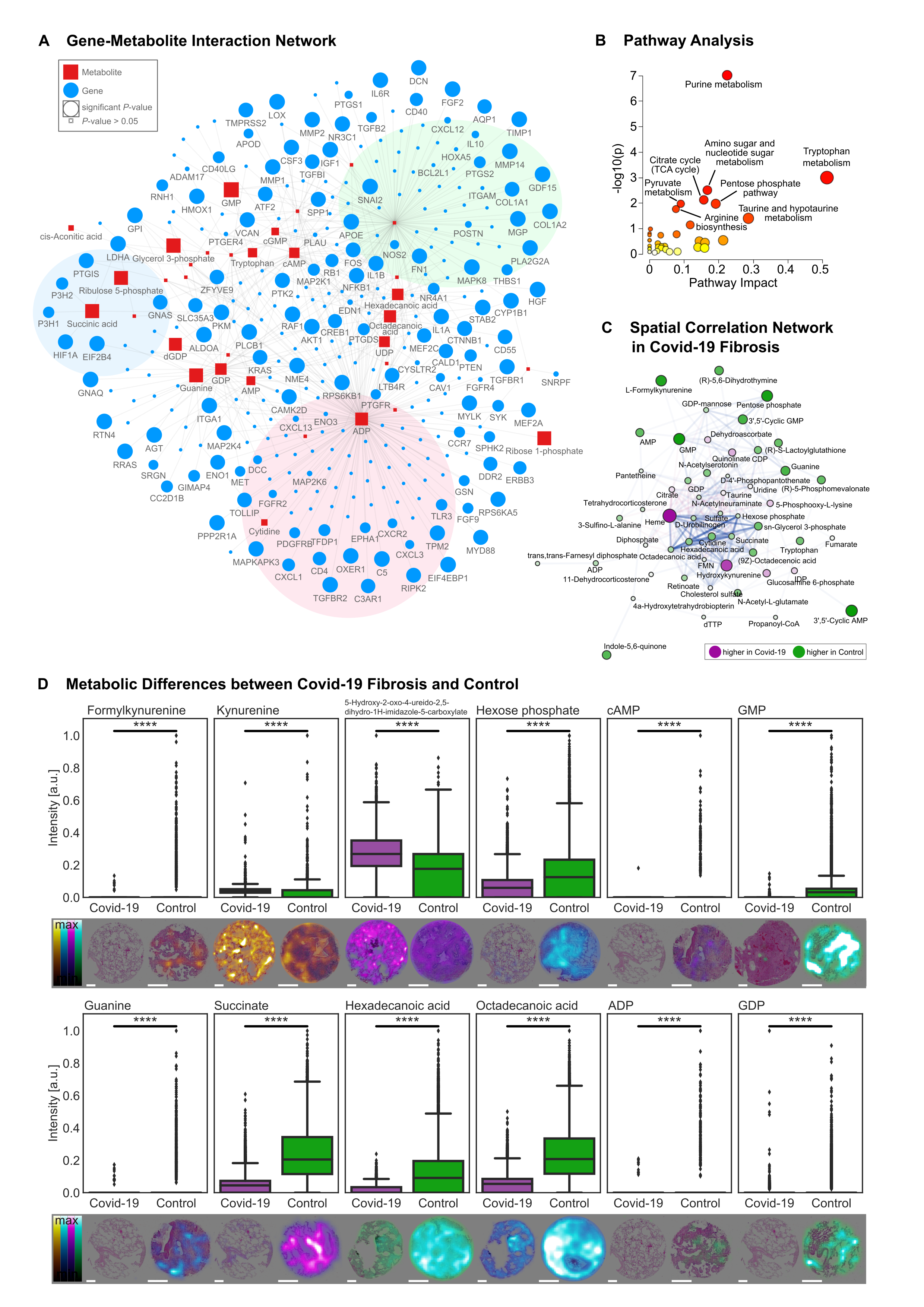

Supplement: Supplementary file 18 [file mmc18.zip › mmc18.tif]
